# The *Salmonella* Typhimurium InvF-SicA complex is necessary for the transcription of *sopB* in the absence of the repressor H-NS

**DOI:** 10.1371/journal.pone.0240617

**Published:** 2020-10-29

**Authors:** Luis E. Romero-González, Deyanira Pérez-Morales, Daniel Cortés-Avalos, Edwin Vázquez-Guerrero, Denisse A. Paredes-Hernández, Paulina Estrada-de los Santos, Lourdes Villa-Tanaca, Miguel A. De la Cruz, Víctor H. Bustamante, J. Antonio Ibarra

**Affiliations:** 1 Laboratorio de Genética Microbiana, Departamento de Microbiología, Escuela Nacional de Ciencias Biológicas, Instituto Politécnico Nacional, Ciudad de México, México; 2 Departamento de Microbiología Molecular, Instituto de Biotecnología, Universidad Nacional Autónoma de México, Cuernavaca, Morelos, México; 3 Unidad de Investigación Médica en Enfermedades Infecciosas y Parasitarías, Hospital de Pediatría, Centro Médico Nacional Siglo XXI, Instituto Mexicano del Seguro Social, Ciudad de México, México; Centre National de la Recherche Scientifique, Aix-Marseille Université, FRANCE

## Abstract

Expression of virulence factors in non-typhoidal *Salmonella enterica* depends on a wide variety of general and specific transcriptional factors that act in response to multiple environmental signals. Expression of genes for cellular invasion located in the *Salmonella* pathogenicity island 1 (SPI-1) is tightly regulated by several transcriptional regulators arrayed in a cascade, while repression of this system is exerted mainly by H-NS. In SPI-1, H-NS represses the expression mainly by binding to the regulatory region of *hilA* and derepression is exercised mainly by HilD. However, the possible regulatory role of H-NS in genes downstream from HilD and HilA, such as those regulated by InvF, has not been fully explored. Here the role of H-NS on the expression of *sopB*, an InvF dependent gene encoded in SPI-5, was evaluated. Our data show that InvF is required for the expression of *sopB* even in the absence of H-NS. Furthermore, in agreement with previous results on other InvF-regulated genes, we found that the expression of *sopB* requires the InvF/SicA complex. Our results support that SicA is not required for DNA binding nor for increasing affinity of InvF to DNA *in vitro*. Moreover, by using a bacterial two-hybrid system we were able to identify interactions between SicA and InvF. Lastly, protein-protein interaction assays suggest that InvF functions as a monomer. Derived from these results we postulate that the InvF/SicA complex does not act on *sopB* as an anti-H-NS factor; instead, it seems to induce the expression of *sopB* by acting as a classical transcriptional regulator.

## Introduction

*Salmonella* species are widely distributed and are the cause of gastroenteritis, diarrhea and typhoid fever around the globe [1,2]. These bacteria have a plethora of virulence factors mainly grouped in discrete genomic regions called *Salmonella* pathogenicity islands (SPIs) [3,4]. SPI-1 and SPI-2 each encode for a type three-secretion system (T3SS), T3SS-1 and T3SS-2, respectively. The T3SS-1 is required for invasion as well as for replication of *Salmonella* in the cytosol of epithelial cells [4–6]. The T3SS-2 is mainly needed for bacterial survival and replication within macrophages, in compartments denoted *Salmonella* containing vacuole (SCV) [4,7].

Expression of T3SS-1 and T3SS-2 has been widely studied in both *in vivo* and *in vitro* conditions, allowing to determine many details of how and when these two virulence apparatuses are expressed. In general, global regulators activate the expression of *Salmonella*-specific transcriptional factors, in response to environmental signals present in the hosts; then, the specific regulators induce the expression of genes required for the colonization of particular *in vivo* niches where *Salmonella* can replicate [8–11]. Among the global regulators, the nucleoid-associated protein H-NS has a pivotal regulatory role in *Salmonella* by acting as a repressor on most virulence genes, including the SPI-1 and SPI-2 regulons [12–17]. Thus, the expression of most *Salmonella* virulence genes is induced by the action of a regulator that counteracts H-NS-mediated repression on the respective promoters [14,18,19].

**Fig 1:**
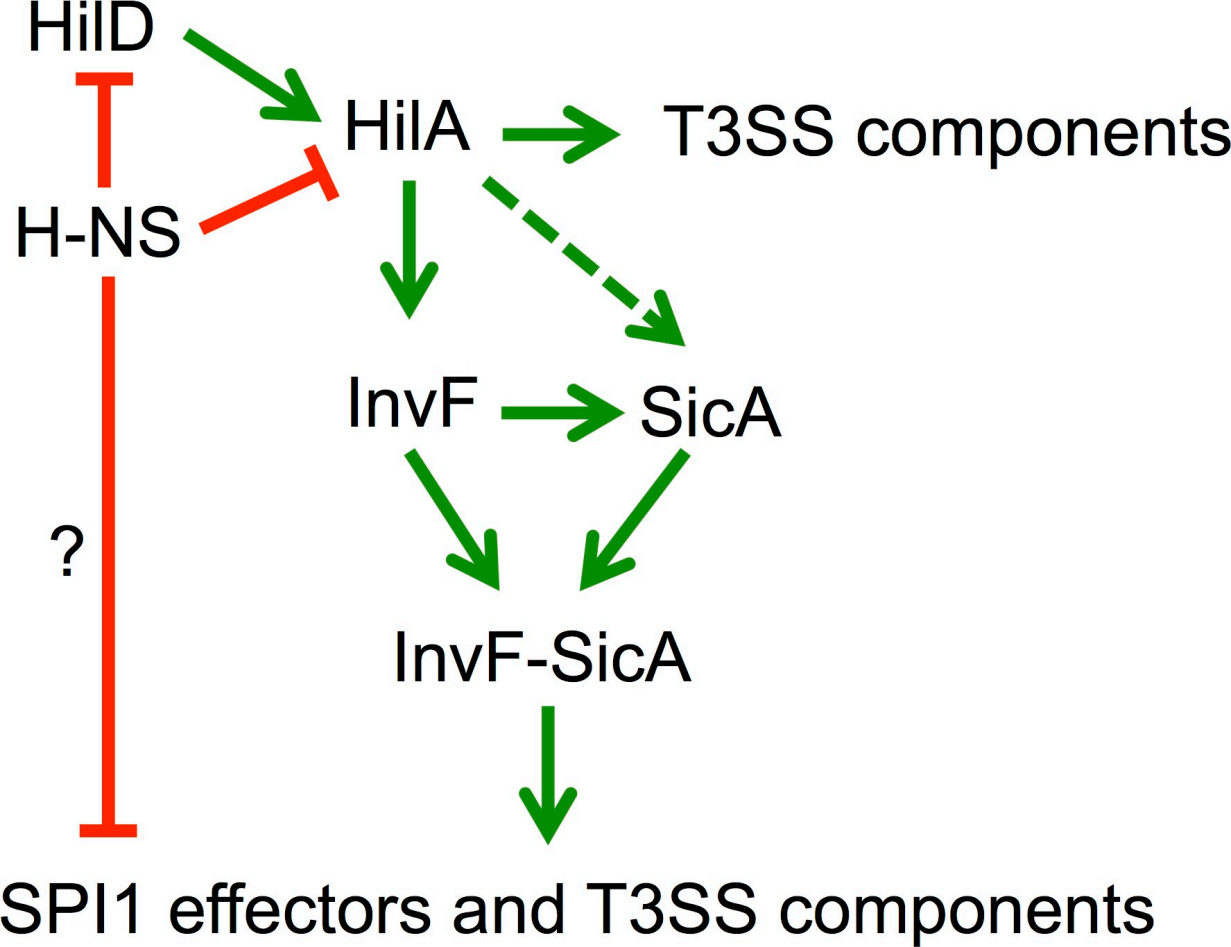
Simplified schematic representation of a section of the SPI-1 regulatory cascade. H-NS represses the expression of HilD and HilA. As described by Ellermeier *et al*. [20], HilD, HilC and RtsA activate HilA transcription but HilD has a predominant role and it is the only one illustrated here. HilD removes H-NS and once HilA is translated it activates genes for T3SS-1 biogenesis and also the last transcriptional regulator in this cascade, InvF. This activates some structural genes and the chaperone SicA. Together, the InvF/SicA complex activates the expression of effector proteins. To our knowledge, it is not known whether H-NS represses the genes coding for the effectors and whether InvF acts as a derepressor or as a classical activator. A green line with an arrow indicates activation of gene expression, a green pointed line indicates readthrough activation and a red blunted line indicates repression of expression.

In regard to SPI-1, this island encodes the HilD, HilC, HilA, InvF and SprB regulators; HilD, HilC and InvF belonging to the AraC/XylS family of transcriptional regulators [8,9]. HilD, HilA and InvF form a regulatory cascade that induces expression of the SPI-1 genes and many other genes located outside SPI-1 (Fig 1). This regulatory cascade starts with a positive feed-forward loop between HilD, HilC and RtsA (encoded outside of the SPI-1); HilD being the dominant transcriptional factor in this regulatory loop [8,20], induces the expression of *hilA* and several other target genes by antagonizing H-NS-mediated repression [8,9,20,21]. Then, HilA activates the expression of genes involved in the biosynthesis of the T3SS-1; additionally, it activates the expression of the InvF transcriptional regulator [22]. Finally, InvF induces the expression of a set of genes encoding effectors that are translocated into host cells through the T3SS-1, including *sopB* (*sigD*), which is located in SPI-5 [23,24] (Fig 1). InvF is the only member of the AraC/XylS family of regulators known to form a complex with another protein to activate its cognate genes [25,26]. That is, in order for InvF to be active it needs to interact with the chaperone protein SicA, which also binds to multiple T3SS-1 components and effectors [27–30]. The InvF/SicA complex positively controls the expression of *sicA*, *sopB*, *sptP*, *sopE*, *sopE2*, *STM1239* and several other genes and, being in the last portion of the regulatory cascade, InvF/SicA controls the timing of effector protein expression [10,28,29]. However, the mechanism by which InvF/SicA induces expression of target genes remains undetermined, i.e. it is unknown if InvF/SicA acts as an anti-H-NS factor, as in the case of HilD, or if it mediates gene expression as a classical transcriptional factor.

Here we show that, in contrast to HilD and other *Salmonella* transcriptional regulators involved in virulence, the InvF/SicA complex is required for the expression of target genes even in the absence of the H-NS repressor. Additionally, we show that InvF binds *in vitro* to the promoter region of *sopB* independently of SicA. However, our results and those previously reported indicate that transcriptional activation of *sopB* requires both InvF and SicA. These findings led us to propose that the InvF/SicA complex might be acting as a classical transcriptional regulator to induce the expression of virulence genes.

## Materials and methods

### Bacterial strains and culture conditions

*Salmonella enterica* serotype Typhimurium SL1344 strain [31] and the isogenic ΔSPI1, ΔSPI1 Δ*rtsA* and *invF*::Tn5 mutants, were previously generated [16,23,32]. Strain SL1344 *invF*::Flag was obtained by transducing the marked gene from strain 14028 *invF*::Flag using P22 phage [33]. *Escherichia coli* MC4100 and its Δ*hns* derivative were used for expression experiments [34,35]. *E*. *coli* DH10b and BL21 (DE3) strains were used for genetic constructs and protein expression, respectively. *E*. *coli* SU101 and SU202 were used for the dimerization experiments [36]. SPI-1 inducing conditions were used as previously described using LB-Miller broth [37]. Briefly, one or two colonies of the *Salmonella* strains to be tested were taken from a fresh plate and inoculated into 3 ml LB-Miller broth (10 g/L tryptone, 5 g/L yeast extract, 10 g/L NaCl, pH 7.0) in a 16 x 100 mm, glass round-bottom test tube with loose cap that was incubated at 37 ^o^C in shaking conditions (220 rpm) for 16 to 18 h. Subsequently, a 300 μl of this culture were subcultured into a 125 ml glass flask containing 10 ml of sterile LB-Miller broth with a loose cap or cotton wool stopper, and incubated at 37 ^o^C in shaking conditions (220 rpm) for 3.5 h (aerated conditions). Media for selecting bacterial clones was LB agar supplemented with ampicillin (100 μg/ml), kanamycin (30 μg/ml), tetracycline (10 μg/ml) or streptomycin (100 μg/ml).

### Construction of plasmids

Plasmids used in this work are listed in S1 Table. To construct a plasmid containing *invF* (pT3-InvF), invF-XH1-Fw and invF-H3-Rv oligos (S1 Table) were used to amplify this gene using *S*. Typhimurium genomic DNA as a template. The PCR product was purified, digested with HindIII and XhoI and then cloned into the pMPM-T3 vector [38]. A plasmid containing *sicA* (pTOPO-SicA) was constructed using the oligos sicA-Fw and sicA-His6-Rv (designed to include a His6 tag) to amplify by PCR *sicA* using *S*. Typhimurium genomic DNA as template. The obtained amplicon was cloned into the pCRTOPO 2.1 vector (Thermo Scientific) using the TOPO-TA cloning kit.

Plasmids pSR658-InvF and pSR658-SicA [39] were constructed by using the oligos lexA-invF-Fw/lexA-invF-Rv2, and sicA-Lex-Fw/sicA-Lex-Rv2 respectively, then the PCR products were purified and digested with XhoI and PstI and cloned into the plasmid pSR658. To construct the plasmid pSR659-SicA, oligos sicA-Lex-Fw and sicA-Lex-Rv3 were used to amplify *sicA*, then the PCR product was digested with XhoI and KpnI and cloned into pSR659. All plasmid constructs were sequenced for verification.

### CAT assays

Chloramphenicol acetyl transferase (CAT) specific activity from the *cat* transcriptional fusions was determined as described before [32,35,40,41]. Briefly, samples of 1 ml were taken from bacterial cultures in SPI-1 inducing conditions described above in this section and in a previous study [37]. Samples were lysed by sonication and soluble extracts were obtained by centrifugation. CAT activity and protein quantification to calculate CAT specific activity were determined for the soluble extracts on 96-well microplates as described previously [32,35,40,41].

### RT-qPCR assays

Relative expression of *sopB* in the different *Salmonella* strains was determined by RT-qPCR as described previously [37,42]. Briefly, RNA was obtained from bacterial cultures grown in SPI-1-inducing conditions. DNA was removed with DNA-Free (Ambion) and then cDNA was obtained with a GoScript kit (Promega). qPCR was performed in a Rotor-gene Q Thermocycler (Qiagen). Relative expression of *sopB* was calculated with the ΔΔCt method using the expression of the gene coding for the 16S rRNA as a normalizer. Oligos for each gene are listed in S1 Table (sopB-RT-Fw and sopB-RT-Rv for *sopB*; Eub338F and Eub518R for the 16S gene). Experiments were done in triplicates and the results are the average of three independent experiments.

### Expression and purification of MBP-InvF and MBP

Both proteins, chimeric protein MBP-InvF and MBP, were purified from *E*. *coli* BL21 harboring plasmids pMal-InvF and pMal-c2xa, respectively, and over-expressed by IPTG induction, lyzed by sonication and by using affinity chromatography with amylose resin as previously described [16,43]. Fractions of the purified proteins were observed in a 12% SDS-PAGE and analyzed by Western blot using anti-MBP antibodies (New England Biolabs). Protein concentrations were determined by following the Bradford protocol and by using an albumin standard curve. In addition to the Western blot analysis, MBP-InvF bands were excised from the Coomassie stained SDS-PAGE and sent for identification by LC-MS/MS to the Centre de Recherche CHU de Québec Proteomics facility. Results were analyzed by using the Scaffold 4 program.

### Expression and purification of SicA-His6

SicA-His6 was purified from cultures of *E*. *coli* BL21 pTOPO-SicA by affinity chromatography with Ni-NTA (Qiagen) as described previously [44,45]. Fractions of the purified protein were observed in a 12% SDS-PAGE and also analyzed by Western blot using an HRP-conjugated His-probe (Thermo Scientific). As a control, the GlpQ-His10 fusion protein previously reported by our lab was also purified [45]. Protein concentration was determined by Bradford with the use of an albumin standard curve.

### Electrophoretic Mobility Shift Assays (EMSAs)

Protein-DNA interactions were observed by a change in the electrophoretic mobility of DNA fragments as described before [43,46]. Briefly, a DNA fragment corresponding to the promoter region of *sopB* was amplified by PCR with the pair of oligos sopB-200-Fw and sopB-Fus-Rv. A region of *fliC* was amplified by PCR with primers fliC-Fw and fliC-Rv and used as a negative control in these assays. Protein molar concentrations were calculated by using the following webpage: http://molbiol.edu.ru/eng/scripts/01_04.html. Proteins and DNA fragments were mixed in 1X binding buffer (10X buffer: 400 mM HEPES, 80 mM MgCl_2_, 500 mM KCl, 10 mM DTT, 0.5% NP40 and 1 mg/ml BSA) [46] to a final volume of 20 to 40 μl and incubated at room temperature for 30 min. DNA fragments were resolved by electrophoresis in a native TBE 6% acrylamide gel and stained with ethidium bromide.

### Protein-protein interactions (pulldown)

Pulldown experiments were performed with purified MBP, MBP-InvF and SicA-His6 proteins. Two mixtures were done: a negative control with MBP and SicA-His6 and tests with MBP-InvF and SicA-His6; both were done by using 50 μg of each protein in an 2X interaction buffer (100 mM NaH_2_PO_4_, 600 mM NaCl, 40 mM imidazol, 0.5% NP-40 and 20% glycerol, pH 8.0) [47]. Proteins were let to interact for 30 min on ice, then 50 μl of amylose resin or Ni-NTA agarose beads were added to each mixture and let to interact for 2 h in agitation at ~4°C. Beads were centrifuged at 2,000 x *g* for 2 min, amylose beads were washed three times with cold washing buffer (see above) and Ni-NTA beads were washed with low imidalozole buffer (Qiagen). After the last washing step, supernatant was removed carefully and then 20 μl of Laemmli buffer were added. Samples were resolved in an SDS-PAGE and stained with Coomassie blue. Western blot was performed by transferring the proteins from the SDS-PAGE to a PVDF membrane (Merck) and by following a previously described protocol [45,47]. Western blot was developed by using chemiluminescence kit (Invitrogen) and observed in a Chemidoc imaging system (Biorad).

Pulldown experiments were also performed with a cell free extract containing InvF-Flag and either MBP or MBP-InvF. For this, *S*. Typhimurium *invF*::Flag strain was transformed with either pMal-InvF or pMal-c2xa, as a negative control. Then, cell free extracts were obtained from bacterial cultures grown in SPI-1-inducing conditions and the addition of 0.3 mM IPTG. One ml of each cell free extract was mixed with 50 μl of amylose resin and let to interact for 2 h in agitation at ~4°C. Beads were centrifuged at 2,000 x *g* for 2 min and washed four times with cold washing buffer (see above). After the last washing step supernatant was removed carefully, 30 μl of Laemmli buffer were added to the beads and samples were boiled for 10 min. Samples were resolved in a SDS-PAGE, Western blot was performed by transferring the proteins to a PVDF membrane (Merck) and using anti-MBP antibodies (New England Biolabs) and anti-FLAG antibodies (Sigma) as suggested by the manufacturers. Membrane developing was done as described above in this section.

### Dimerization assays

A LexA-based two hybrid system was used to evaluate protein-protein interactions between InvF and SicA [36,39]. In order to verify the integrity of the LexA-derived proteins a Western blot was performed by using the primary antibody anti-LexA (Millipore) (1;2,000) and the secondary antibody anti-rabbit IgG-HRP (Sigma-Aldrich) (1;10,000) (S4 Fig). To test InvF and SicA homodimerization competent cells of *E*. *coli* SU101 were transformed with pSR658-InvF or pSR658-SicA and selected in LB plates with the corresponding antibiotic (S1 Table). Selected transformants were grown in LB supplemented with 1 mM IPTG and let them grow to an OD of 0.6. Aliquots were taken to assess β-galactosidase activity as described before [48]. To test InvF and SicA heterodimerization, *E*. *coli* SU202 was co-transformed with the plasmids pSR658-InvF and pSR659-SicA and tested as described above. The plasmids pSR658-HNS, pSR658-HilD and pSR659-HilE were used as controls in the experiments [49].

### Statistical analysis

Statistical analysis was performed in Excel (Microsoft) or GraphPad Prism version 5 (GraphPad Software) by using a Student’s t-test. A significant difference was considered when *P* < 0.05.

## Results

### The InvF/SicA complex does not act as an anti-HNS factor on *sopB*

**Fig 2:**
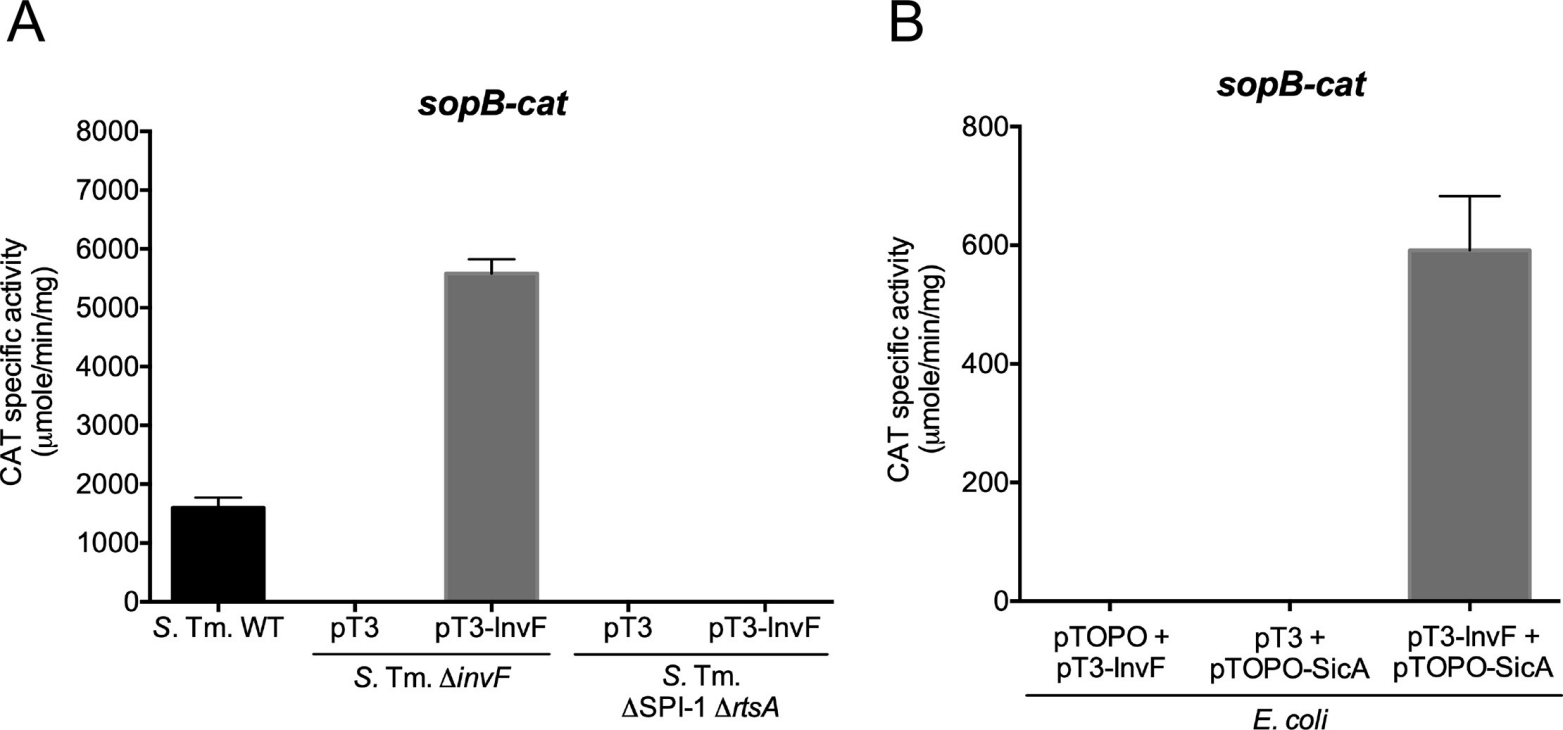
InvF and SicA are required for expression of *sopB*. *sopB* expression in *Salmonella* Typhimurium (A) and *E*. *coli* (B) strains was determined by means of a transcriptional fusion to the *cat* reporter gene (psopB-cat1) in SPI-1 inducing conditions, as mentioned in the Methods section. The pT3-InvF and pTOPO-SicA plasmids constitutively express InvF and SicA, respectively, and the corresponding empty vectors are indicated (pT3 is pMPM-T3 and pTOPO is pCR2.1TOPO-TA, see S1 Table). Bars represent the average of at least three different experiments and error bas are standard deviations.

The global transcriptional regulator H-NS has been shown to play an important role as a repressor of the expression of many virulence genes in *Salmonella* [8,9,20,21] (Fig 1). As for genes depending on InvF, several evidences have shown that this regulator, together with SicA, is necessary for expression [28,29] (Fig 1). Here we questioned whether InvF induces gene expression by antagonizing H-NS-mediated repression on target genes, for which *sopB* was used as a probe gene. Our reasoning was that if H-NS represses *sopB* and InvF just acts as an anti-H-NS factor, then the expression of this gene should be induced in the absence of H-NS independently of InvF similarly as it has been observed with other virulence genes in *Salmonella*. To further investigate the action mechanism of InvF, we first analyzed the activity of a *sopB-cat* transcriptional fusion in the WT *S*. Typhimurium strain and its isogenic Δ*invF* mutant carrying the pT3-InvF plasmid, which expresses InvF from a constitutive promoter, or carrying the pMPM-T3 vector. As expected, the *sopB-cat* fusion was not expressed in the *invF* mutant; furthermore, its activity was restored in the *invF*::*Tn5* mutant by the presence of the pT3-InvF plasmid, to levels even higher than those reached by this fusion in the WT strain (Fig 2A).

Additionally, since previous studies indicate that InvF needs to interact with the SicA chaperone to be active [28,29], we analyzed the activity of the *sopB-cat* fusion in a *Salmonella* strain lacking SPI-1 and RtsA (ΔSPI1 Δ*rtsA*). As expected, the pT3-InvF plasmid was not able to induce expression of *sopB-cat* in this mutant strain (Fig 2A). Similar results were observed when an *Escherichia coli* K-12 surrogate system was used. Expression of *sopB-cat* was only induced when both the pT3-InvF plasmid expressing InvF and the pTOPO-SicA plasmid expressing SicA constitutively, were present (Fig 2B). These results corroborate previous reports showing that expression of *sopB* requires the InvF/SicA complex.

**Fig 3:**
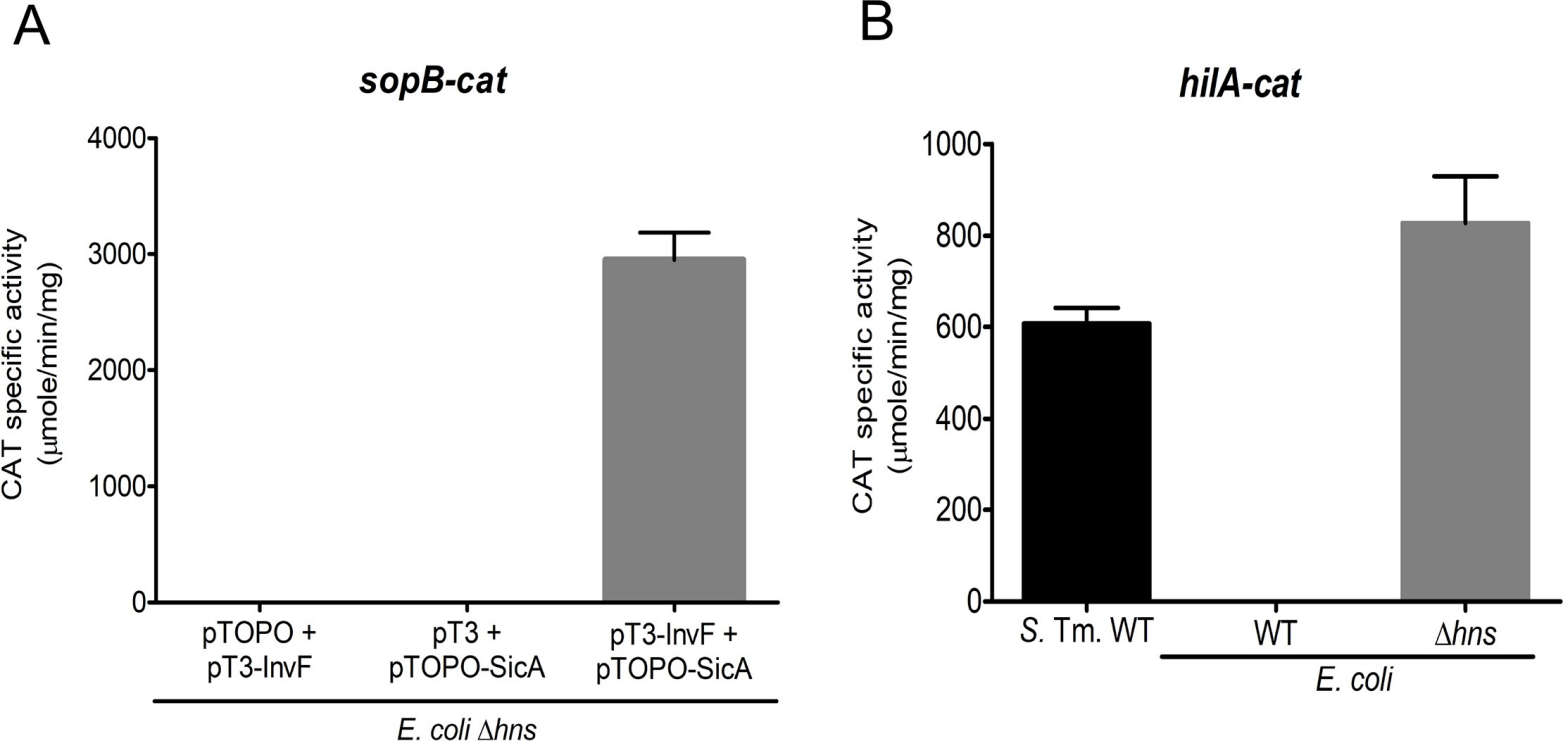
*sopB* expression is dependent of InvF/SicA in an *E*. *coli hns* mutant. *sopB* (A) and *hilA* (B) expression was determined in wt and *Δhns E*. *coli* strains using transcriptional fusions to *cat* (psopB-cat1 and philA-cat-410+66, respectively) in SPI-1 inducing conditions as mentioned in the Methods section. The pT3-InvF and pTOPO-SicA plasmids constitutively express InvF and SicA, respectively and the corresponding empty vectors are indicated (pT3 is pMPM-T3 and pTOPO is pCR2.1TOPO, see S1 Table). Bars represent the average of at least three different experiments and error bas are standard deviations.

Then, the effect of the inactivation of H-NS on the activity of the *sopB-cat* fusion was analyzed when the InvF/SicA complex is not present. Ideally, a *Salmonella hns* mutant should be used for these experiments, but this mutation has been shown to be unstable because it causes the overexpression of SPI-1 and other related genes, and suppressor mutations are generated [50]. Thus, we decided to use a surrogate system, an *E*. *coli* Δ*hns* mutant, which has been useful to study the effect of H-NS on other *Salmonella* virulence genes [51]. As a positive control, the *hilA-cat* transcriptional fusion was assessed in WT *E*. *coli* and its derivative Δ*hns* mutant; H-NS represses expression of *hilA* [52,53]. As seen in Fig 3, while the expression of the *hilA-cat* transcriptional fusion was induced in the *E*. *coli* Δ*hns* mutant (Fig 3B), the expression of *sopB-cat* was not (Fig 3A). As observed in the WT *E*. *coli* strain, the *sopB-cat* fusion was expressed in the *E*. *coli* Δ*hns* mutant only in the presence of both InvF and SicA (Fig 3A). These results confirm previous reports made in *Salmonella* indicating that both InvF and the chaperon SicA are required *in vivo* for expression of InvF-dependent genes and that both proteins are necessary for expression of *sopB* even in the absence of the global repressor H-NS when using an *E*. *coli* surrogate system.

### SicA is not required for InvF binding to the *sopB* promoter

**Fig 4:**
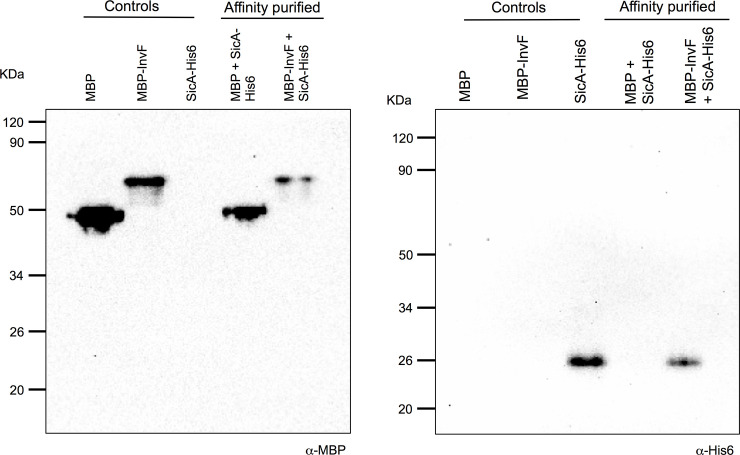
InvF interacts with SicA *in vitro*. Purified versions of MBP, MBP-InvF and SicA-His6 in the combinations: MBP + SicA-His6 and MBP-InvF + SicA-His6, were let to interact in solution and then pulled down with amylose resin. Interactions were detected by Western blot with anti-MBP (A) or anti-His6 (B) (labeled “Affinity purified”). Proteins alone are shown in the first three lanes of each gel as controls and the interactions are indicated.

**Fig 5:**
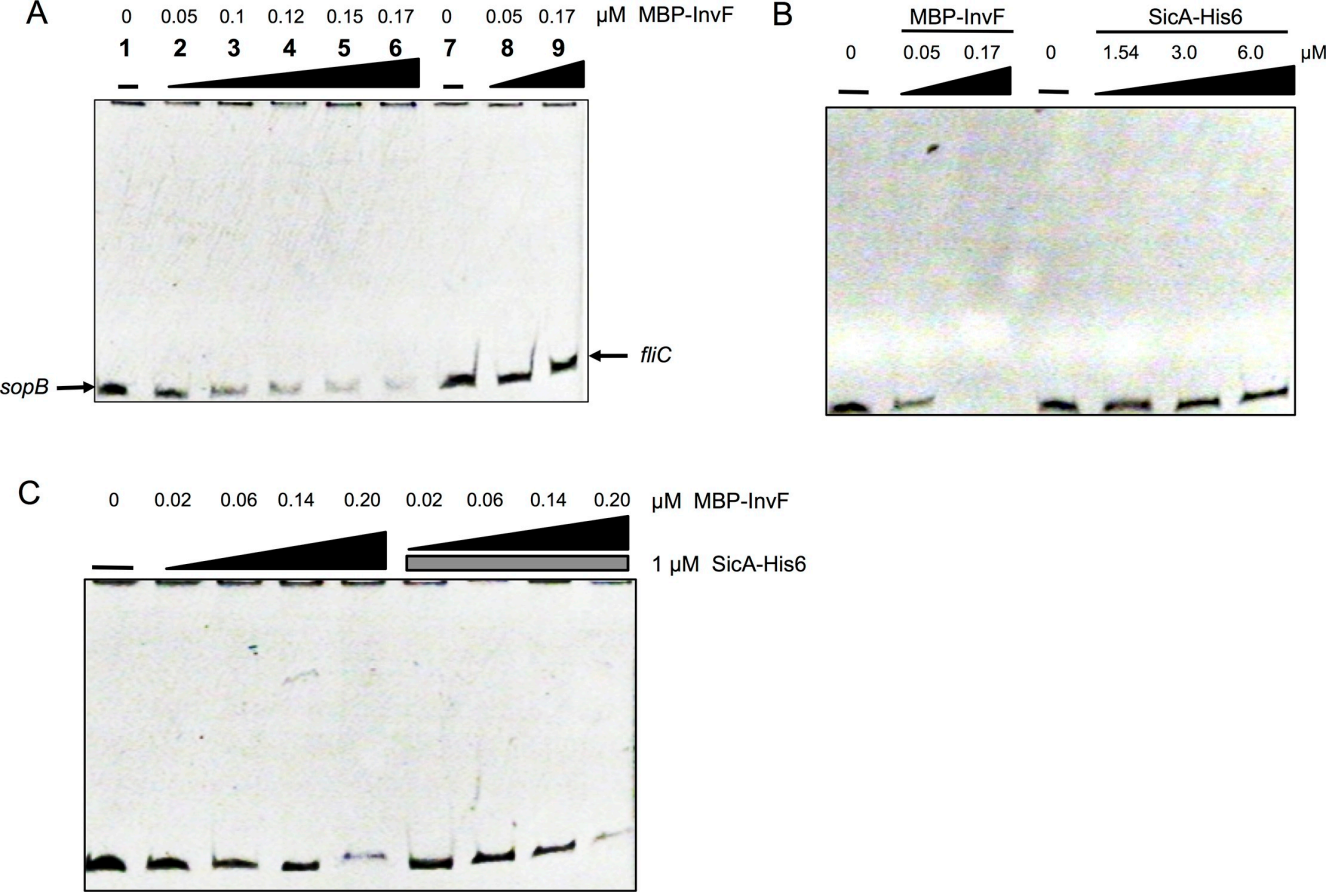
MBP-InvF interaction with *sopB* regulatory region is not dependent on SicA. EMSA experiments were performed with purified MBP-InvF or SicA-His6 or both with the regulatory regions of *sopB*. (A and B) Increasing amounts of MBP-InvF protein were used as shown. Increasing amounts of SicA-His6 protein and MBP-InvF proteins were used as shown in the image. (C) In order to determine the effect of both proteins in DNA binding, increasing amounts of MBP-InvF (dark triangle) and constant amount of SicA-His6 (grey rectangle) were used. Protein-DNA complexes were resolved by electrophoresis in native acrilamyde gels and stained with ethidium bromide. Images were reverted to improve visualization.

To find possible interactions between InvF and the *sopB* regulatory region the MBP-InvF protein was used for electrophoretic motility shift assays (EMSAs). First, in order to determine whether this construct encoding the protein fusion was able to complement a *Salmonella invF*::Tn5 mutant *sopB* mRNA levels were measured and the result showed that indeed it was able to partially complement the mutation (S1 Fig). In order to corroborate the interaction between the tagged versions of InvF and SicA both proteins were purified by using their corresponding tags: MBP and His6, respectively (S2 and S3 Figs). Both proteins were purified but MBP-InvF was very unstable once purified protein fractions were collected; multiple lower bands were observed in the collected fractions that contained the MBP tag (S1B Fig). A band corresponding to the expected size for the MBP-InvF fusion (~ 77 kDa) and those underneath this one were excised and analyzed through LC-MS/MS. Peptides corresponding mainly to MBP (MALE_ECOLI, P0AEX9) and a few corresponding to InvF (P69343) were detected. These data suggest that the MBP-InvF fusion protein is being degraded after purification. Despite these issues, the two purified proteins were used to detect interactions by pull down experiments (Fig 4). Both proteins were able to interact with each other by either using amylose, as a negative control purified MBP was not able to interact with SicA-His6. This result further corroborates previous results showing that InvF interacts with SicA [29,54] and shows that proteins used in this study are functional. Later, this interaction was further corroborated with a bacterial two-hybrid system (see below). Next, MBP-InvF was tested for its ability to bind to the *sopB* regulatory region comprised between -200 to +79 with respect to the transcriptional start site (Fig 5A). As a negative control, a DNA fragment of a similar size from the coding region of *fliC* was also assessed. MBP-InvF was able to bind to the *sopB* DNA fragment and it did not shift the *fliC* fragment. In these experiments we were unable to detect DNA-protein complexes in the gel, but the disappearance of free DNA was observed only with the *sopB* regulatory region and not with a non-related DNA fragment (Fig 5A). Some faint bands were observed but they were not clear and were also observed in the lanes without protein, so these were discarded. These results showed that MBP-InvF is able to bind specifically to the *sopB* regulatory region and suggests that SicA is not needed for this role *in vitro*.

Next we asked whether SicA is able to independently bind to DNA. For this, increasing amounts of SicA-His6 were used with a DNA fragment containing the *sopB* regulatory region. As seen in Fig 5B, a DNA shift was not observed even when a large amount of protein was used, while a control DNA shift with MBP-InvF required a low amount of protein. To explore the possibility that SicA increases MBP-InvF affinity for its binding site, both proteins were tested *in vitro* (Fig 5C). Results showed that an increase in MBP-InvF affinity for the *sopB* regulatory region was not observed. Altogether, these results support that InvF binds *in vitro* to the regulatory region of *sopB* independently of SicA.

### InvF does not form dimers

**Fig 6:**
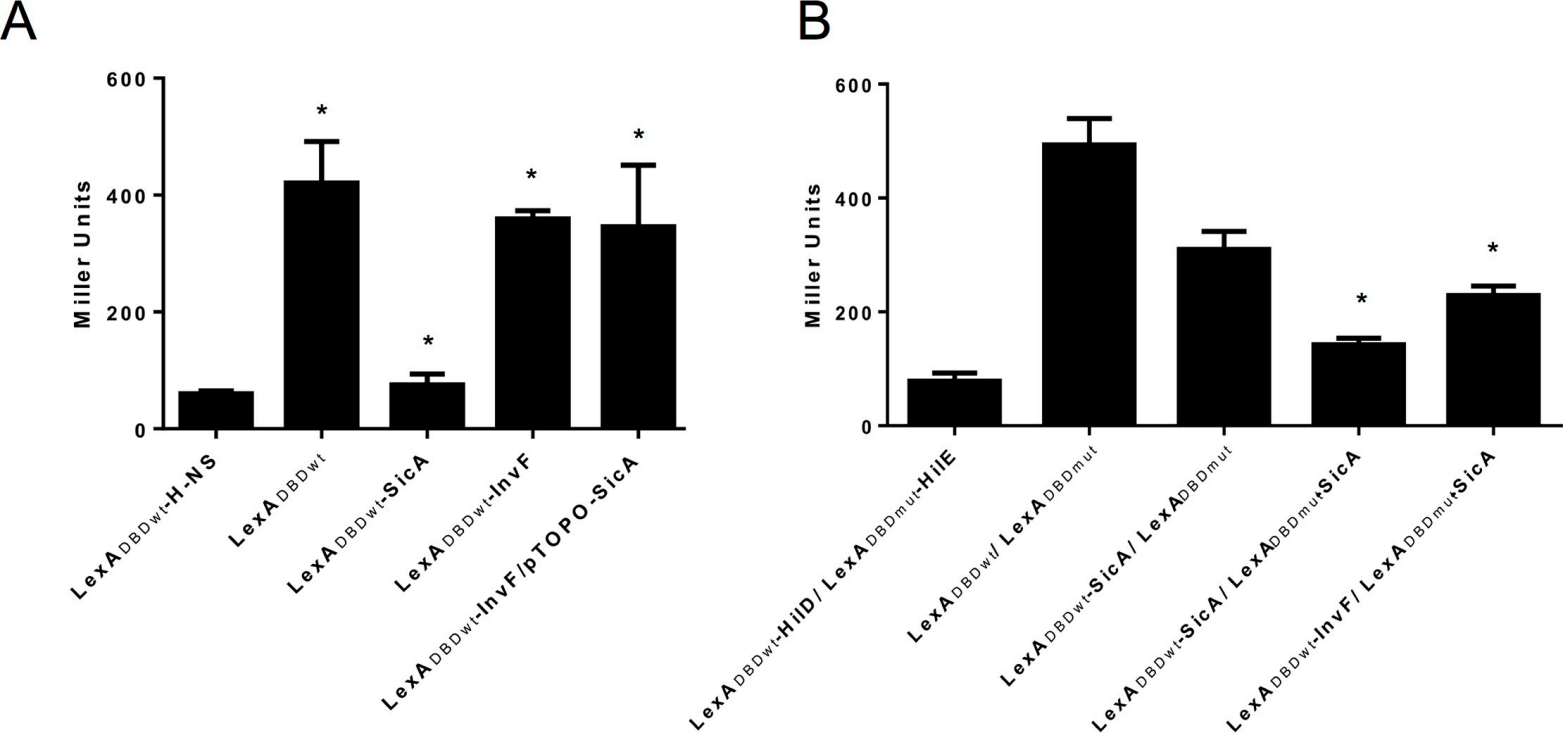
LexA-based two-hybrid analysis of InvF and SicA interactions. LexA-derivatives were introduced either in strain SU101 (A) or SU202 (B) and grown and processed as described in the Methods section. Constructs or combinations of them are indicated below each bar. Bars represent the average of at least three independent experiments and the error bars represent the standard deviation. *, indicates statically significance difference (*P* < 0.01) as follows: For (A), LexA_DBDwt_-H-NS vs. LexA_DBDwt_; LexA_DBDwt_-SicA vs.LexA_DBDwt_; LexA_DBDwt_-InvF vs. LexA_DBDwt_-H-NS; and LexA_DBDwt_-InvF/pTOPO-SicA vs. LexA_DBDwt_-H-NS; For (B) LexA_DBDwt_/LexA_DBDmut_ vs. LexA_DBDwt_-SicA/LexA_DBDmut_-SicA; and LexA_DBDwt_/LexA_DBDmut_ vs. LexA_DBDwt_-InvF/LexA_DBDmut_-SicA.

Some members of the AraC/XylS family of transcriptional regulators, such as AraC, HilD, etc., are able to form dimers both in solution and when binding to their target DNA [25,49]; dimerization is, in some cases, important for the role as a transcriptional regulator. Mutants defective in this dimer formation are not able to either repress or activate. In order to determine whether InvF is able to form dimers, a bacterial LexA based two-hybrid system was used [39,49]. In this system the LexA DNA-binding domain (LexA_DBDwt_) is fused to the protein of interest and then tested with a *sulA-lacZ* chromosomal transcriptional fusion. When there is no interaction there is β-galactosidase activity; in contrast, when there is protein-protein interaction then there is no enzymatic activity from the reporter fusion [36]. As shown in Fig 6A this system demonstrated to be useful to detect SicA dimerization; in contrast, InvF did not form dimers. Considering the possibility that SicA affects InvF stability or that the chaperone was needed for InvF to form dimers, SicA was supplied in *trans*. Results showed that even when SicA was present dimer formation for InvF was not detected. One possibility was that LexA_DBDwt_ coupled to InvF impedes the InvF-SicA interaction. In order to test this latter, the interaction between InvF and SicA was tested when both proteins are fused to LexA_DBDwt_ and LexA_DBDmut_ in the heterodimer system. As observed in Fig 6B, control tests using HilD and HilE and also SicA-SicA showed interactions as expected. When InvF and SicA were tested the expression of the reporter fusion was also diminished, demonstrating an interaction among these proteins. Unexpectedly, LexA_DBDwt_-SicA combined with the empty vector encoding LexA_DBDmut_ also showed a lower activity than that compared to the HilD-HilE control but it was not as low as that observed with the SicA-SicA control and with InvF-SicA. Thus, our results suggest that InvF does not form dimers.

**Fig 7:**
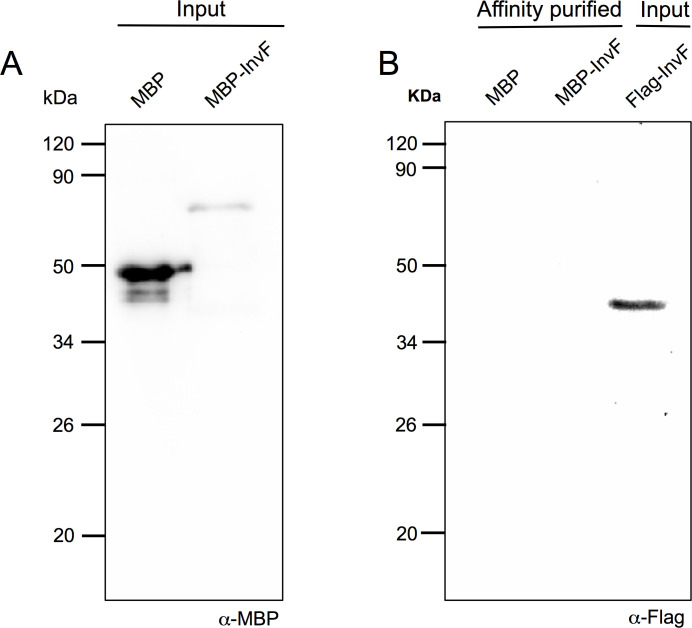
InvF does not form dimers. Plasmids expressing either MBP or MBP-InvF were transformed into a *Salmonella invF*::Flag strain and samples were collected from IPTG and SPI-1 induced cultures. Cell free extracts were prepared, pulled down with amylose resin to detect interactions of MBP and MBP-InvF and tested by Western blot. In (A) pull downs were tested to detect production of both MBP versions with an anti-MBP antibody (Input). In (B) an anti-Flag antibody was used to detect possible interactions of InvF-Flag with MBP and MBP-InvF (Affinity purified). As a control for the expression of InvF-Flag in the tested conditions the last lane shows a whole cell extract (Input).

In order to corroborate that InvF is not able to interact with itself the pMal-InvF plasmid was transformed in a *Salmonella invF*::Flag strain. Once induction was reached by adding IPTG in SPI-1 inducing conditions, cells were obtained and sonicated to obtain a cellular extract which was used for a pulldown experiment with amylose resin. Results showed that MBP-InvF did not interact with the genomic InvF-Flag version (Fig 7B). Together, these results demonstrate that InvF does not form dimers and suggest that it is likely acting as a monomer.

## Discussion

Expression of the SPI-1 virulence genes requires the concerted action of several regulators, including InvF and HilD, both encoded in SPI-1 [8,9,20]. In this regulatory cascade HilD induces the expression of these genes mainly by antagonizing H-NS mediated repression, which means that in the absence of H-NS the SPI-1 genes are expressed independently of HilD (Fig 1) [12,13,14,16,51]. In this work, by analyzing the regulation of the *sopB* gene by InvF, we further define the mechanism by which InvF induces expression of target genes. First, our results further confirm that InvF needs to act together with the SicA chaperone to be active and that both proteins interact *in vitro*. Additionally, we found that the InvF/SicA complex is required for the expression of *sopB* even in the absence of H-NS, which supports that InvF/SicA acts as a classical transcriptional activator. Classical activators recruit the RNA polymerase to the promoters they activate, while derepressors remove the negative effect of a repressor [19,55,56]. Alternatively, InvF/SicA could act on *sopB* by antagonizing repression mediated by an unknown factor, other than H-NS.

In agreement with our results indicating that InvF does not act as an anti-H-NS factor on *sopB*, we previously demonstrated that purified H-NS does not bind to the *sopB* regulatory region [16,51]. Moreover, ChIP-on-chip experiments by Lucchini *et al*. [12] and Navarre *et al*. [13] did not show a significant signal for binding of H-NS to the *sopB* promoter nor they showed overexpression of *sopB* in the absence of H-NS. These findings strongly suggest that H-NS is not directly involved in the regulation of *sopB*. In any case, what it is clear from our results is that the InvF/SicA complex is requiered to initiate the transcription of *sopB* in the absence of H-NS. This contrasts with other SPI-1 and SPI-2 regulated genes in which H-NS plays a repressor role that is removed under inducing conditions by dedicated transcriptional regulators [12,13,18,51].

Our data further corroborates the previously shown dimerization of SicA [29,57] and the interaction between InvF and SicA described by Darwin and Miller [29] and Kim *et al*. [54]. Here, in contrast to results reported by Darwin and Miller [29], we were able to observe the InvF-SicA interaction using a bacterial two-hybrid system based on LexA. Though this interaction was not as clear as that observed for the SicA-SicA interaction, InvF-SicA interaction was corroborated as mentioned above. Moreover, results using pulldown experiments and the LexA-based system have shown that InvF is not able to form homodimers, indicating that it acts as a monomer. In general, the transcriptional regulators in the AraC/XylS family form dimers, but it is not uncommon that some of them act as monomers. This is the case for PerA and Rns [43,58], which are two classical regulators involved in the activation of virulence genes in *E*. *coli*. Previously, Darwin and Miller [29] suggested a putative binding site for InvF which overlaps the suggested -35 box in the regulatory regions of *sicA*, *sopB* and *sopE*. Moreover, they showed experimentally that the proposed sequence in *sicA* is indeed a binding site for InvF. The length of these putative binding sites suggests that only one monomer is able to bind. Thus, it is possible that only one InvF molecule binds to it as we suggest. Ideally, in order to precisely define the InvF binding sites a footprinting should be done; in this case Darwin and Miller did a point-mutation analysis for the *sicA* promoter region and corrobotrated that the InvF binding site was indeed the one they suggested [29]. This latter supports the idea that InvF binds as a monomer to its biding site and, given the fact that this site overlaps with the -35 site, it is likely that it makes contact with the RNA polymerase. This possibility is currently being tested in our laboratory.

Here, in order to observe the ability of InvF to bind to DNA a chimeric MBP-InvF protein was used. AraC/XylS regulators are very unstable as documented elsewhere, but previous reports from our labs and others have shown that MBP stabilizes these proteins [43,59,60]. One of the initial steps was to demonstrate that the addition of MBP to InvF was not a hindrance for the regulator protein. When using *sopB* as a probe, we observed that its expression was almost recovered when complementing an *invF* mutant with a plasmid encoding the chimera. Then, by using the purified MBP-InvF protein our results indicate that it can bind to the regulatory region of *sopB* in the absence of SicA, which is consistent with a previous report showing InvF binding to *sicA* regulatory region independently of SicA [29].

Our data also confirms that SicA does not bind to DNA. Moreover, when MBP-InvF and SicA-His6 were tested in combination for binding on *sopB*, an increase in the affinity of InvF was not detected. Given that SicA is needed *in vivo* for InvF function we cannot discard the likelihood that an increase in DNA affinity happens when it is expressed in *Salmonella*. Darwin and Miller [29] also reported that the role of SicA does not increase InvF stability; however, we observed that purified MBP-InvF is unstable as it seems to be degraded even in the presence of commercial proteinase inhibitors.

## Conclusions

In summary, our study supports the idea that the InvF/SicA complex could act as a classical transcriptional activator and not as an anti-H-NS factor. Additionally, our results suggest that InvF acts as a monomer in a similar fashion as other AraC/XylS regulators that are also classical regulators.

## Supporting information

S1 Fig Plasmid encoding MBP-InvF complements an invF::Tn5 mutant. Expression of *sopB* was detected by qRT-PCR using the gene coding for rRNA 16S as a normalizer. Indicated strains were grown in SPI-1 inducing conditions and samples were taken for RNA extraction. Bars represent the average of at least three independent experiments and the error bars represent +/- SD. **, indicates statically significance difference (*P* < 0.05).
(TIFF)

S2 Fig MBP-InvF purification. MBP-InvF was purified as described in the Methods section and fractions of this process were taken and observed in a 12% SDS-PAGE (A). Lanes: 1, non-induced cells; 2, IPTG-induced cells; 3–8, eluted fractions obtained from the amylose column. (B) Immune detection (WB) of MBP (lane 1) and MBP-InvF (lanes 2–4) with an anti-MBP antibody. Lines in both panels indicate variants of MBP-InvF.
(TIFF)

S3 Fig SicA-His6 purification. SicA-His6 was purified and samples of this process were taken and observed in a 12% SDS-PAGE (A). Lanes: 1, non-induced cells; 2, IPTG-induced cells; 3–8, eluted fractions obtained from the Ni-NTA column. (B) Immune detection (WB) using a HRP-His probe: Control protein GlpQ-His10 (lane 1), non-induced cells (lane 2), and eluted fractions (3–6).
(TIFF)

S4 Fig Integrity of LexA-derived versions of InvF and SicA. Samples of the indicated proteins were obtained after inducing with IPTG and subjected to electrophoresis. Proteins were transfered to PVDF and developed using an anti-LexA antibody as described in the Methods section. Controls included the non-fused wild type LexA DNA binding domain encoded in pRS658 (LexA_DBDwt_), LexA_DBDwt_ fused to repressor H-NS, and non-fused mutated LexA DNA binding domain encoded in pRS659 (LexA_DBDmut_) [39]. *, indicates the expected LexA_DBDwt_ or LexA_DBDmut_ fusion product.
(TIFF)

S1 Table Plasmids and oligonucleotides used in this study. (DOCX)

S1 Raw files (ZIP)

S1 Raw pictures (PDF)
