# Supplementary figures and images for: The Salmonella Typhimurium InvF-SicA complex is necessary for the transcription of sopB in the absence of the repressor H-NS
Source: PLoS One. 2020 Oct 29;15(10):e0240617. doi: 10.1371/journal.pone.0240617 (PMC7595419; doi:10.1371/journal.pone.0240617)

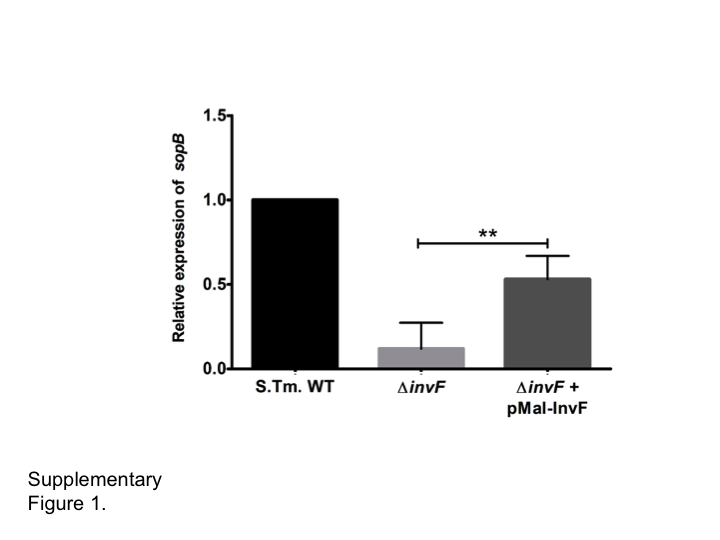

Supplement: S1 Fig — Expression of sopB was detected by qRT-PCR using the gene coding for rRNA 16S as a normalizer. Indicated strains were grown in SPI-1 inducing conditions and samples were taken for RNA extraction. Bars represent the average of at least three independent experiments and the error bars represent +/- SD. **, indicates statically significance difference (P < 0.05). (TIFF) [file pone.0240617.s001.tiff]

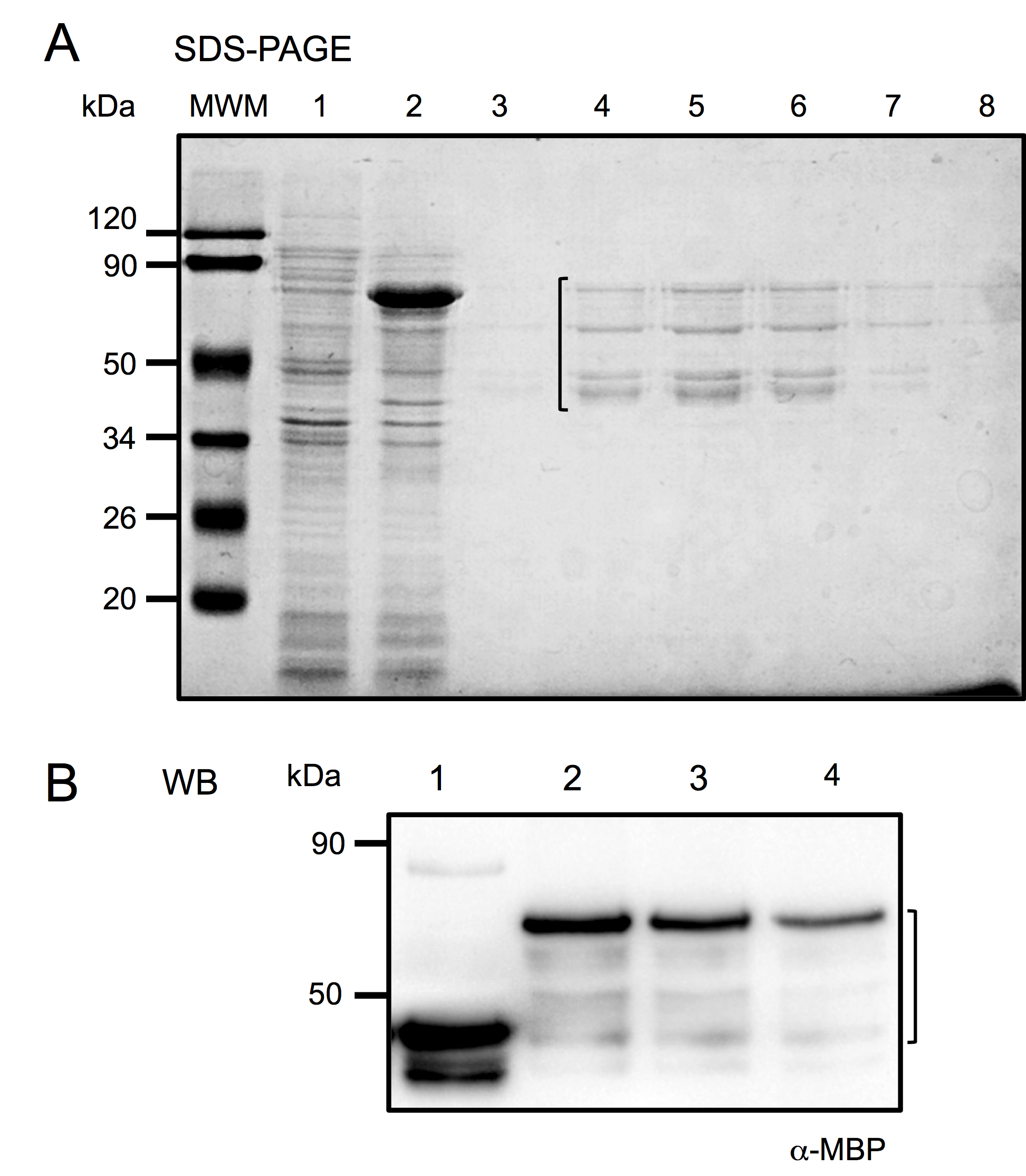

Supplement: S2 Fig — MBP-InvF was purified as described in the Methods section and fractions of this process were taken and observed in a 12% SDS-PAGE (A). Lanes: 1, non-induced cells; 2, IPTG-induced cells; 3–8, eluted fractions obtained from the amylose column. (B) Immune detection (WB) of MBP (lane 1) and MBP-InvF (lanes 2–4) with an anti-MBP antibody. Lines in both panels indicate variants of MBP-InvF. (TIFF) [file pone.0240617.s002.tiff]

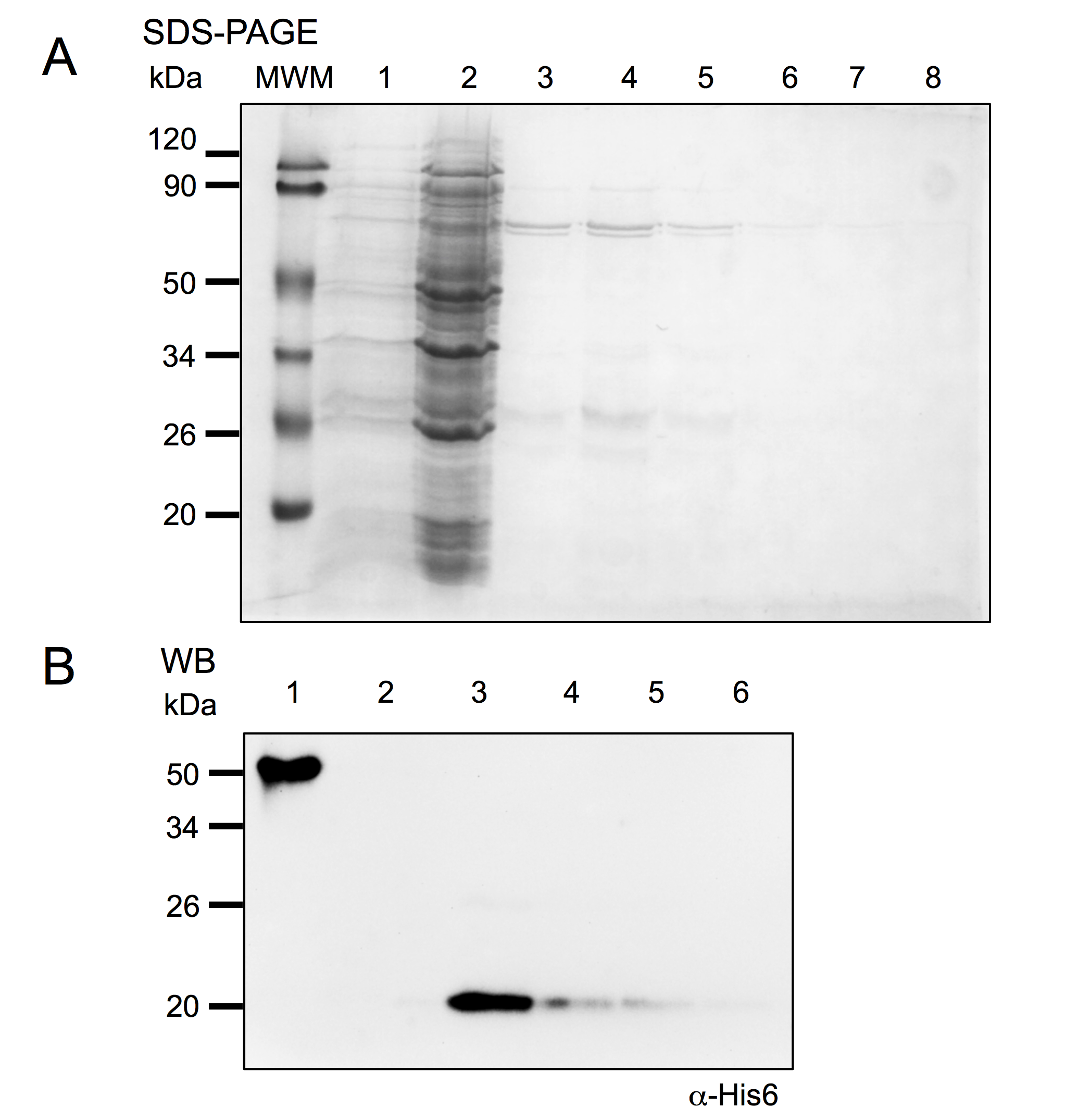

Supplement: S3 Fig — SicA-His6 was purified and samples of this process were taken and observed in a 12% SDS-PAGE (A). Lanes: 1, non-induced cells; 2, IPTG-induced cells; 3–8, eluted fractions obtained from the Ni-NTA column. (B) Immune detection (WB) using a HRP-His probe: Control protein GlpQ-His10 (lane 1), non-induced cells (lane 2), and eluted fractions (3–6). (TIFF) [file pone.0240617.s003.tiff]

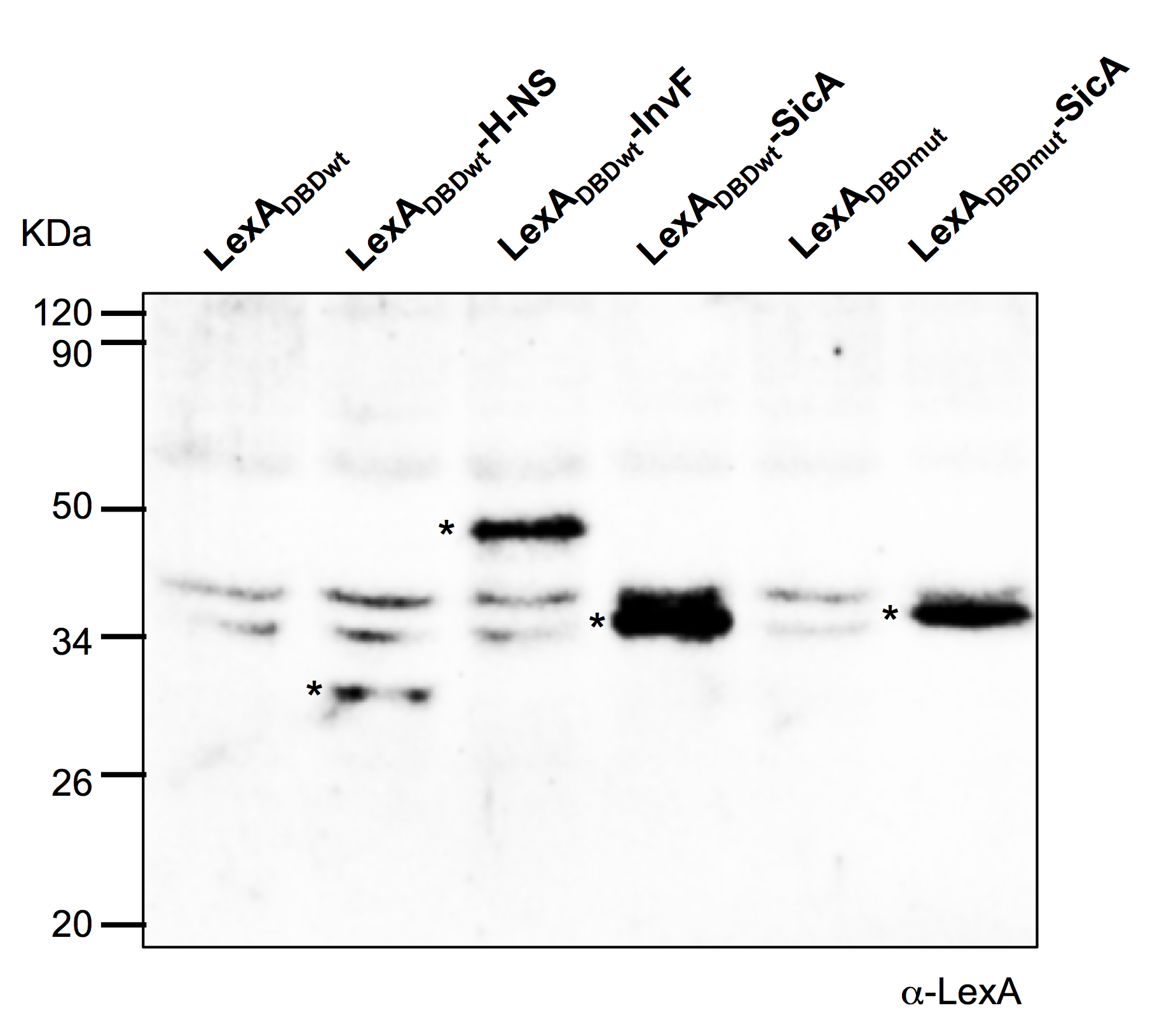

Supplement: S4 Fig — Samples of the indicated proteins were obtained after inducing with IPTG and subjected to electrophoresis. Proteins were transfered to PVDF and developed using an anti-LexA antibody as described in the Methods section. Controls included the non-fused wild type LexA DNA binding domain encoded in pRS658 (LexADBDwt), LexADBDwt fused to repressor H-NS, and non-fused mutated LexA DNA binding domain encoded in pRS659 (LexADBDmut) [39]. *, indicates the expected LexADBDwt or LexADBDmut fusion product. (TIFF) [file pone.0240617.s004.tiff]

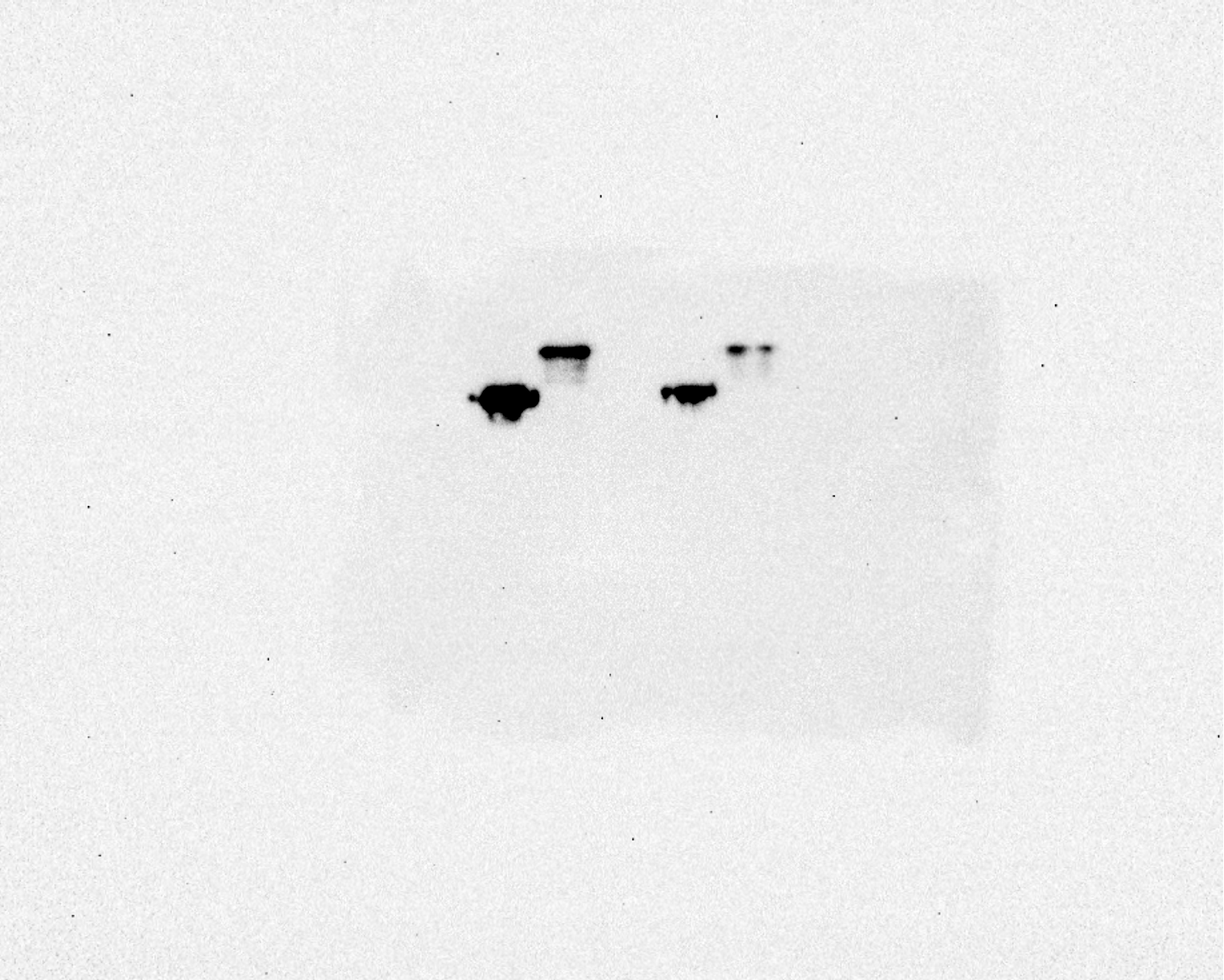

Supplement: S1 Raw files — (ZIP) [file pone.0240617.s006.zip › Archivos Raw/Figura 4A.tif]

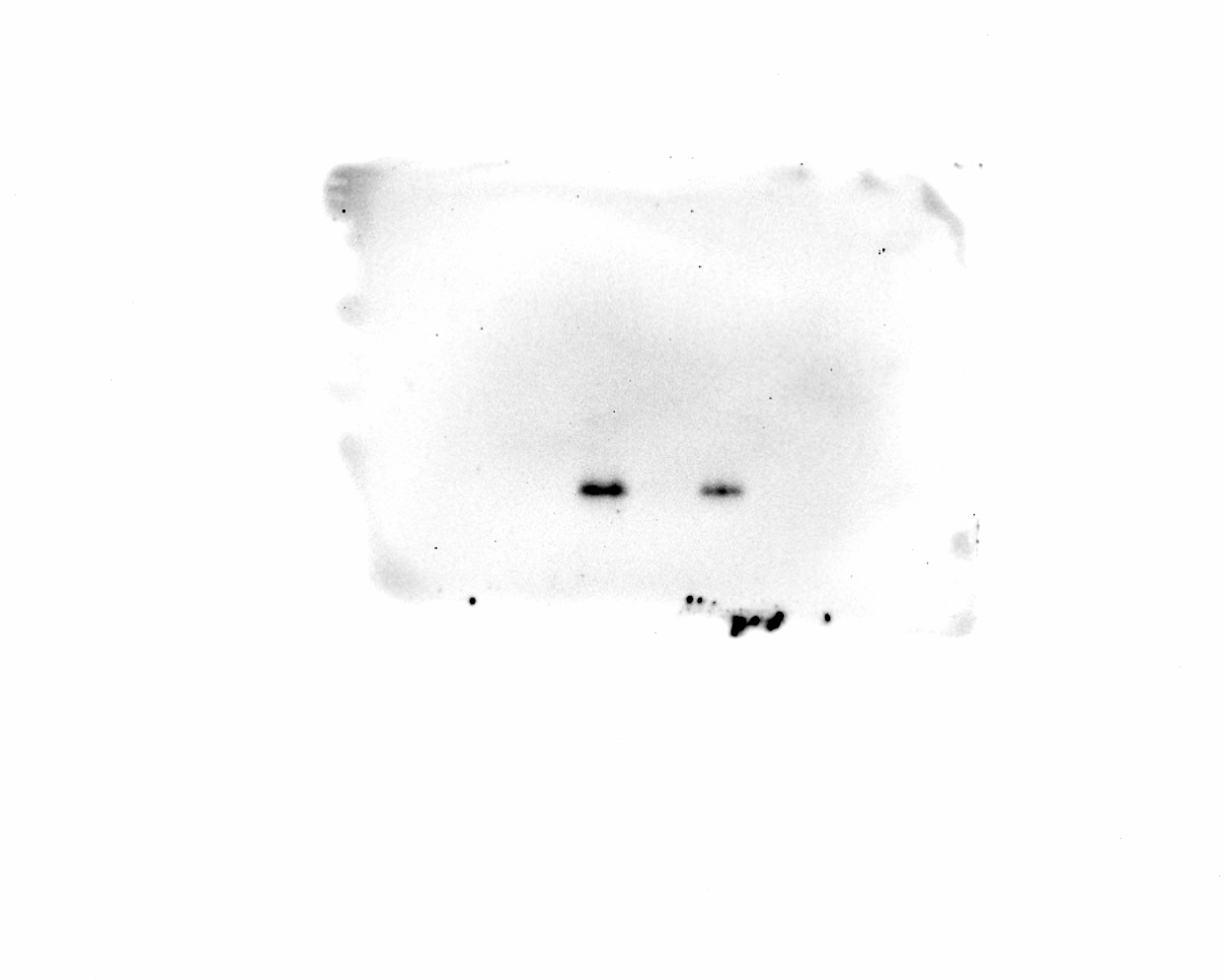

Supplement: S1 Raw files — (ZIP) [file pone.0240617.s006.zip › Archivos Raw/Figura 4B.tif]

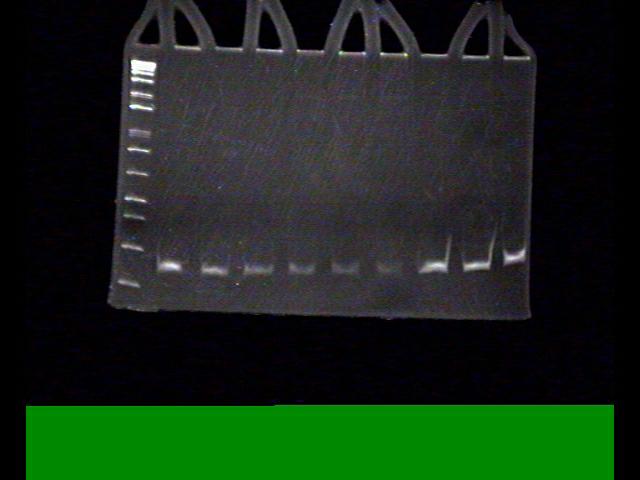

Supplement: S1 Raw files — (ZIP) [file pone.0240617.s006.zip › Archivos Raw/Figure5A.jpg]

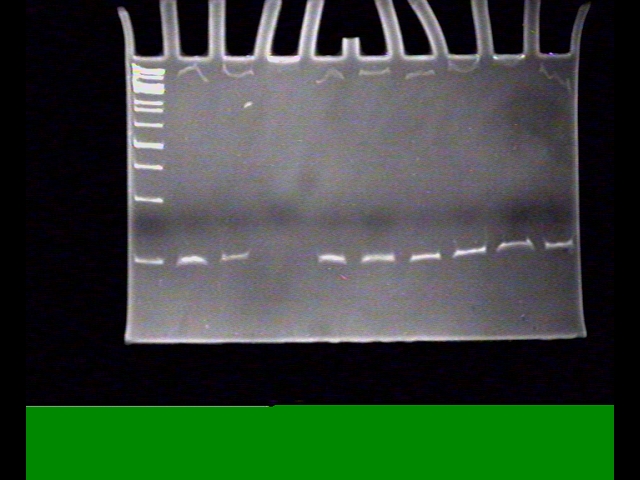

Supplement: S1 Raw files — (ZIP) [file pone.0240617.s006.zip › Archivos Raw/Figure5B.jpeg]

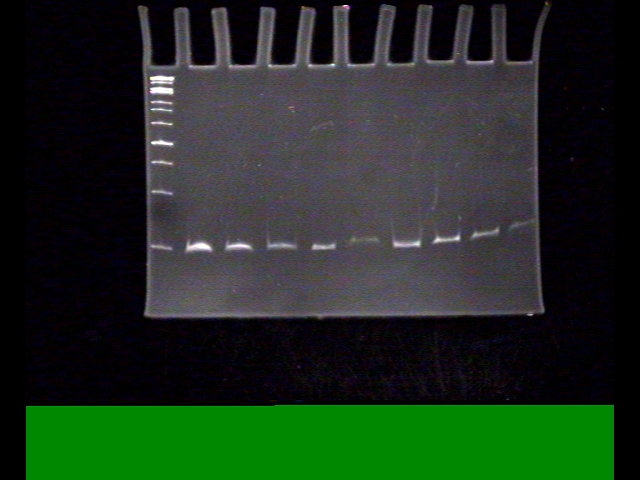

Supplement: S1 Raw files — (ZIP) [file pone.0240617.s006.zip › Archivos Raw/Figure5C.jpg]

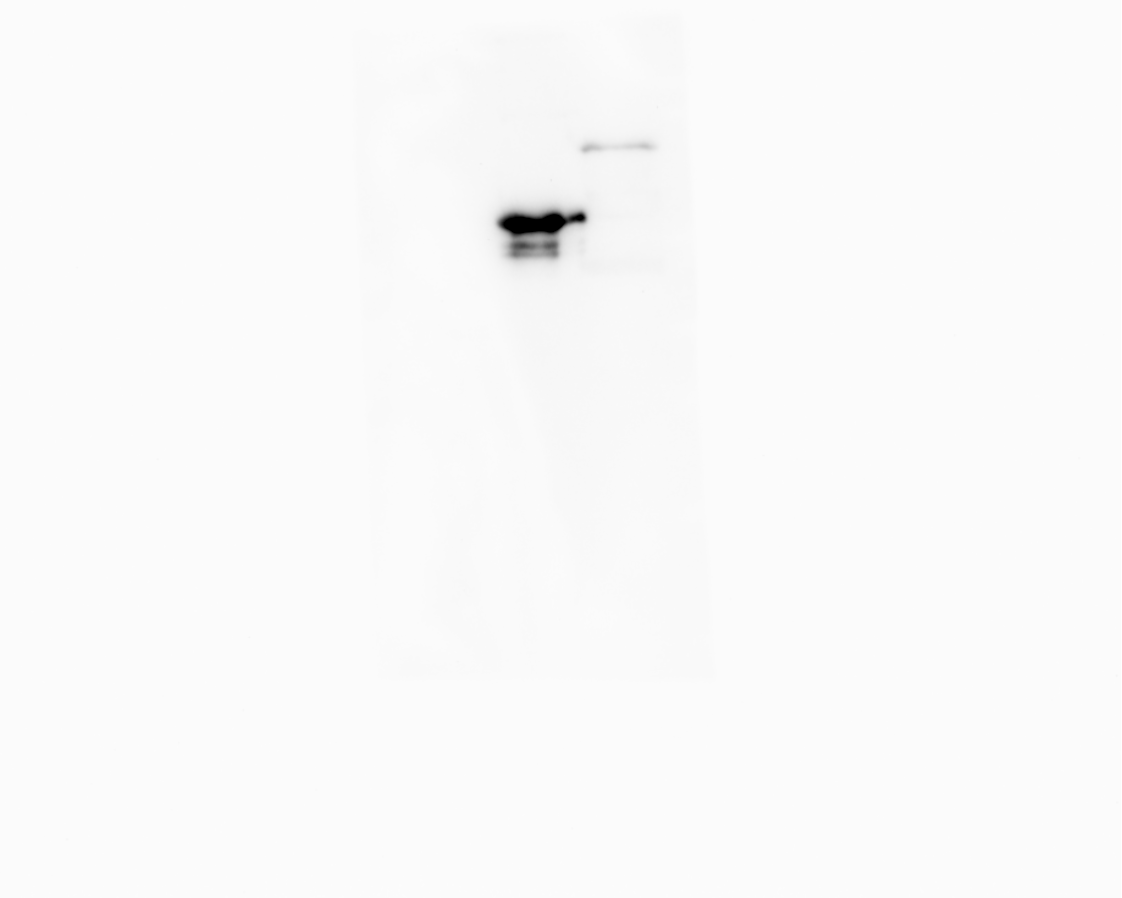

Supplement: S1 Raw files — (ZIP) [file pone.0240617.s006.zip › Archivos Raw/Figure7A.tif]

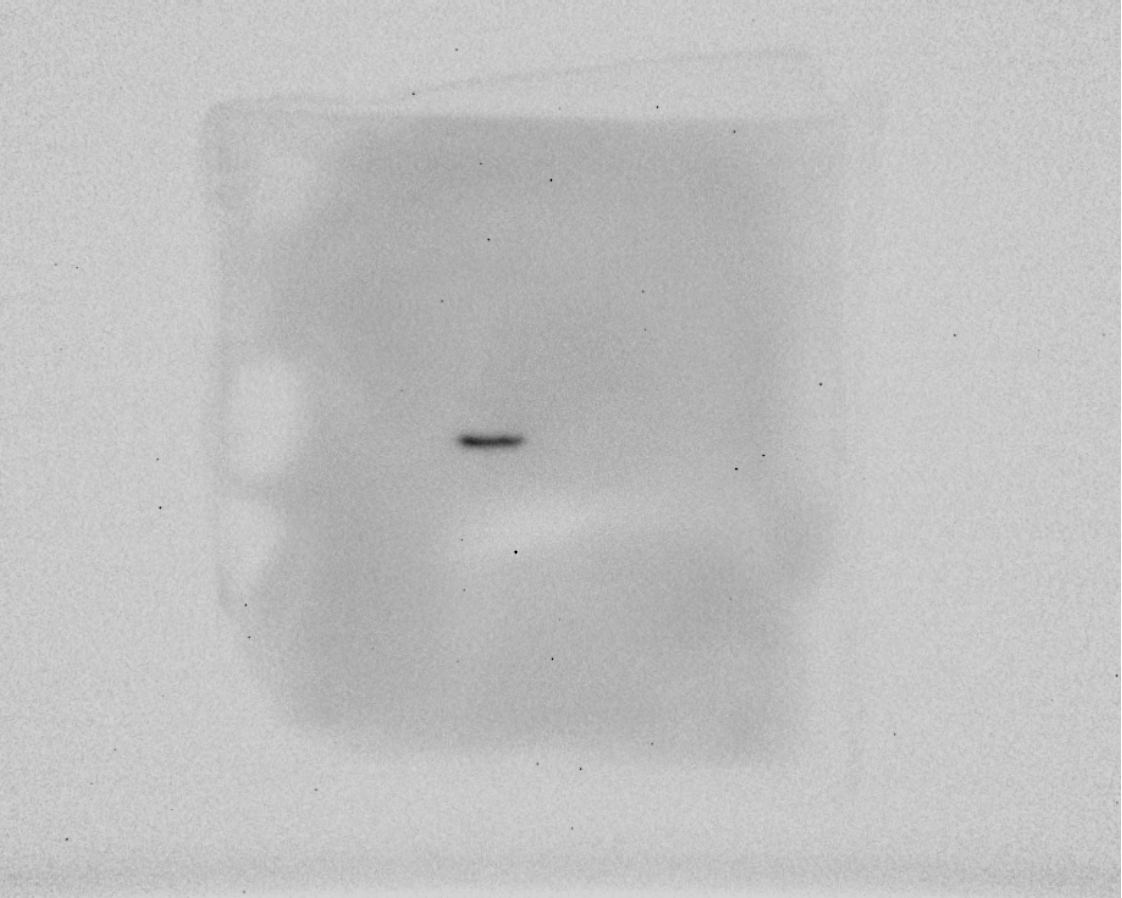

Supplement: S1 Raw files — (ZIP) [file pone.0240617.s006.zip › Archivos Raw/Figure7B.tif]

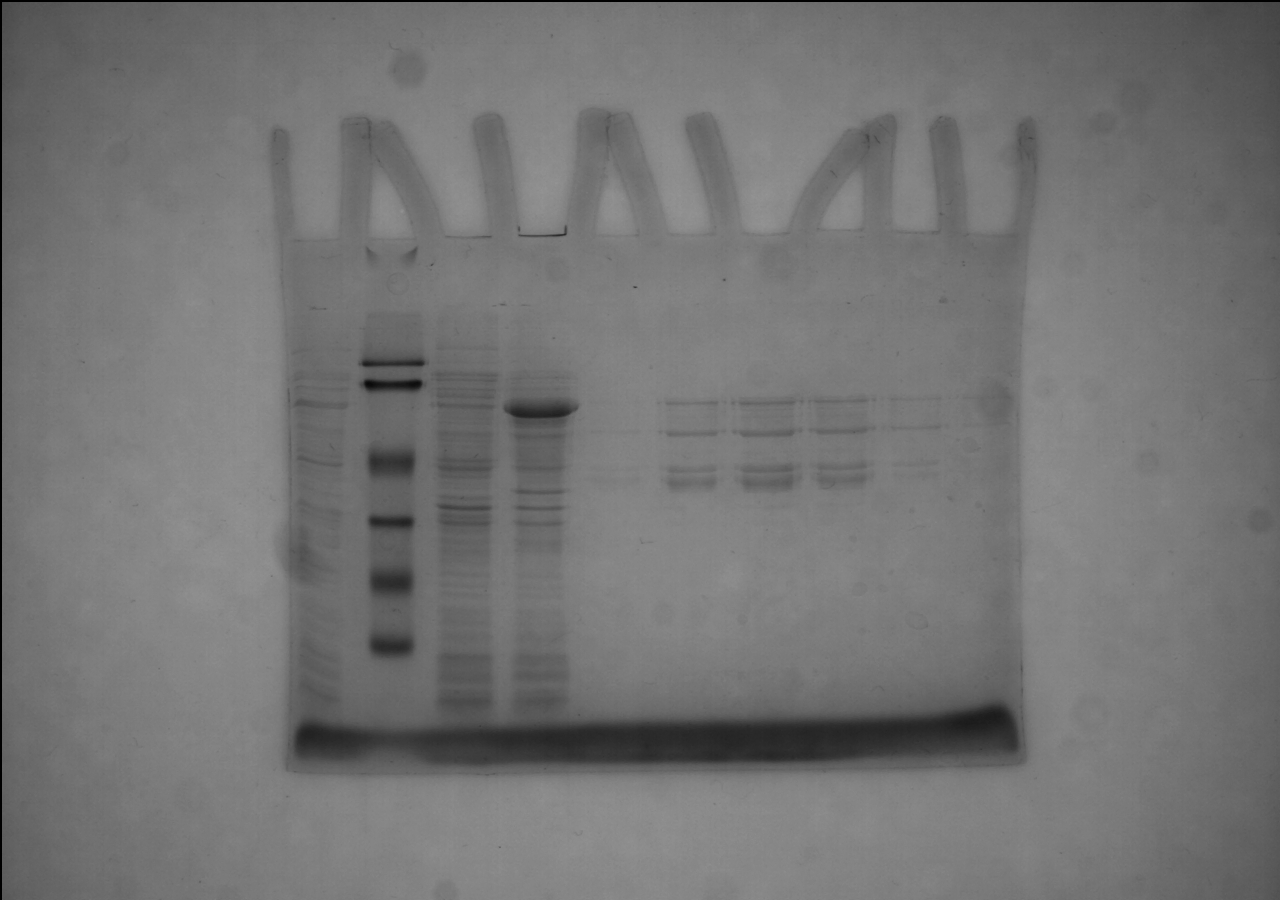

Supplement: S1 Raw files — (ZIP) [file pone.0240617.s006.zip › Archivos Raw/SFigure2A.jpeg]

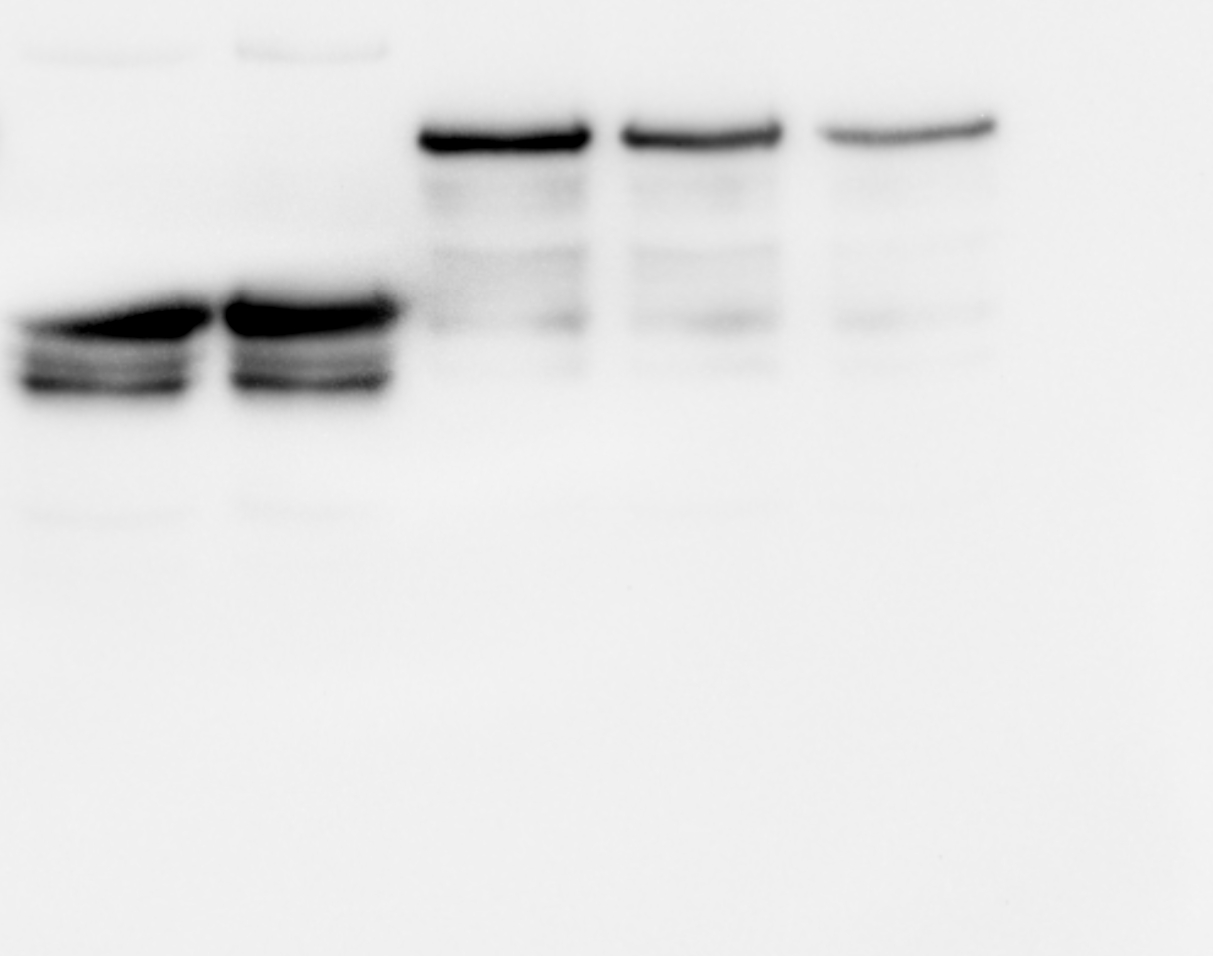

Supplement: S1 Raw files — (ZIP) [file pone.0240617.s006.zip › Archivos Raw/SFigure2B.jpg]

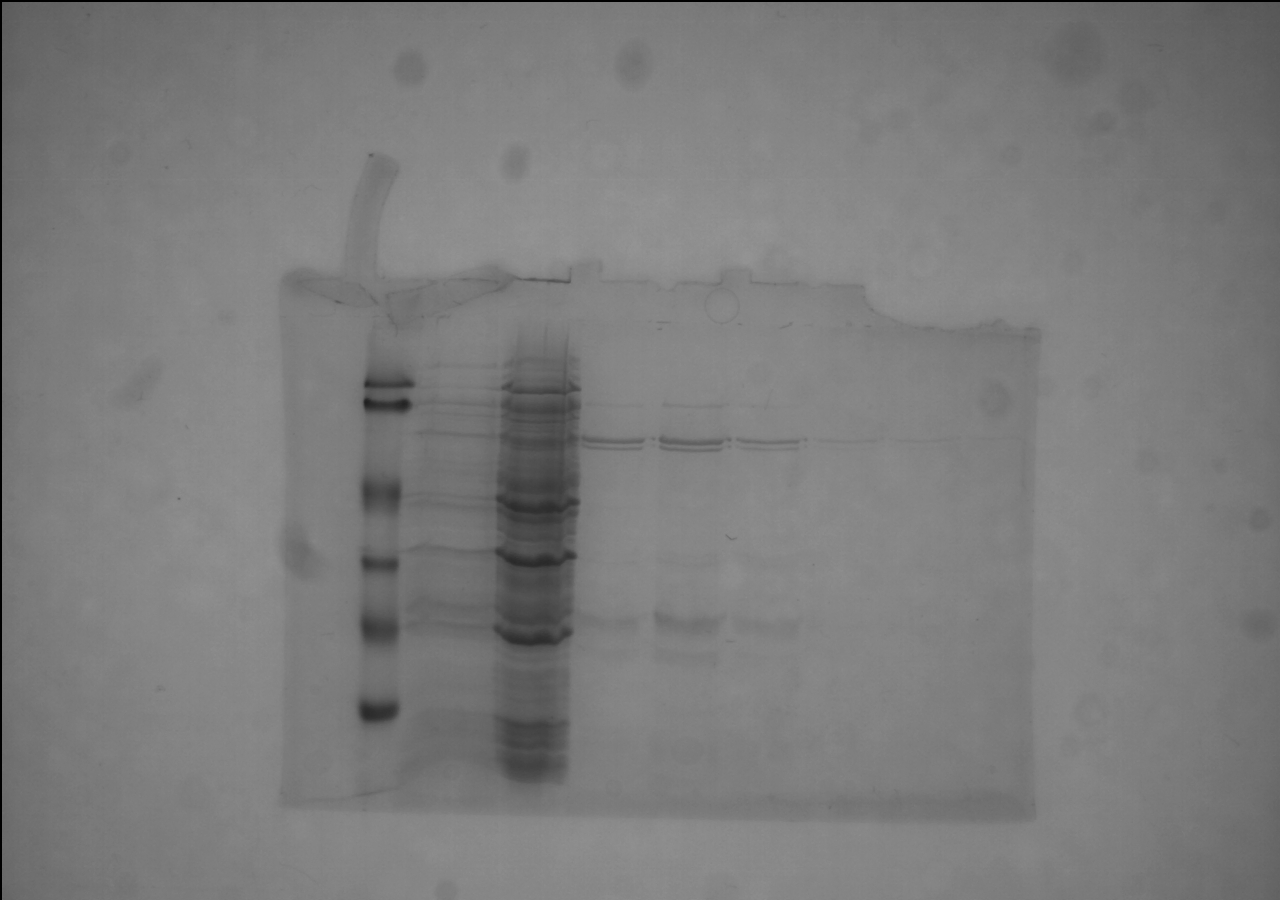

Supplement: S1 Raw files — (ZIP) [file pone.0240617.s006.zip › Archivos Raw/SFigure3A.jpg]

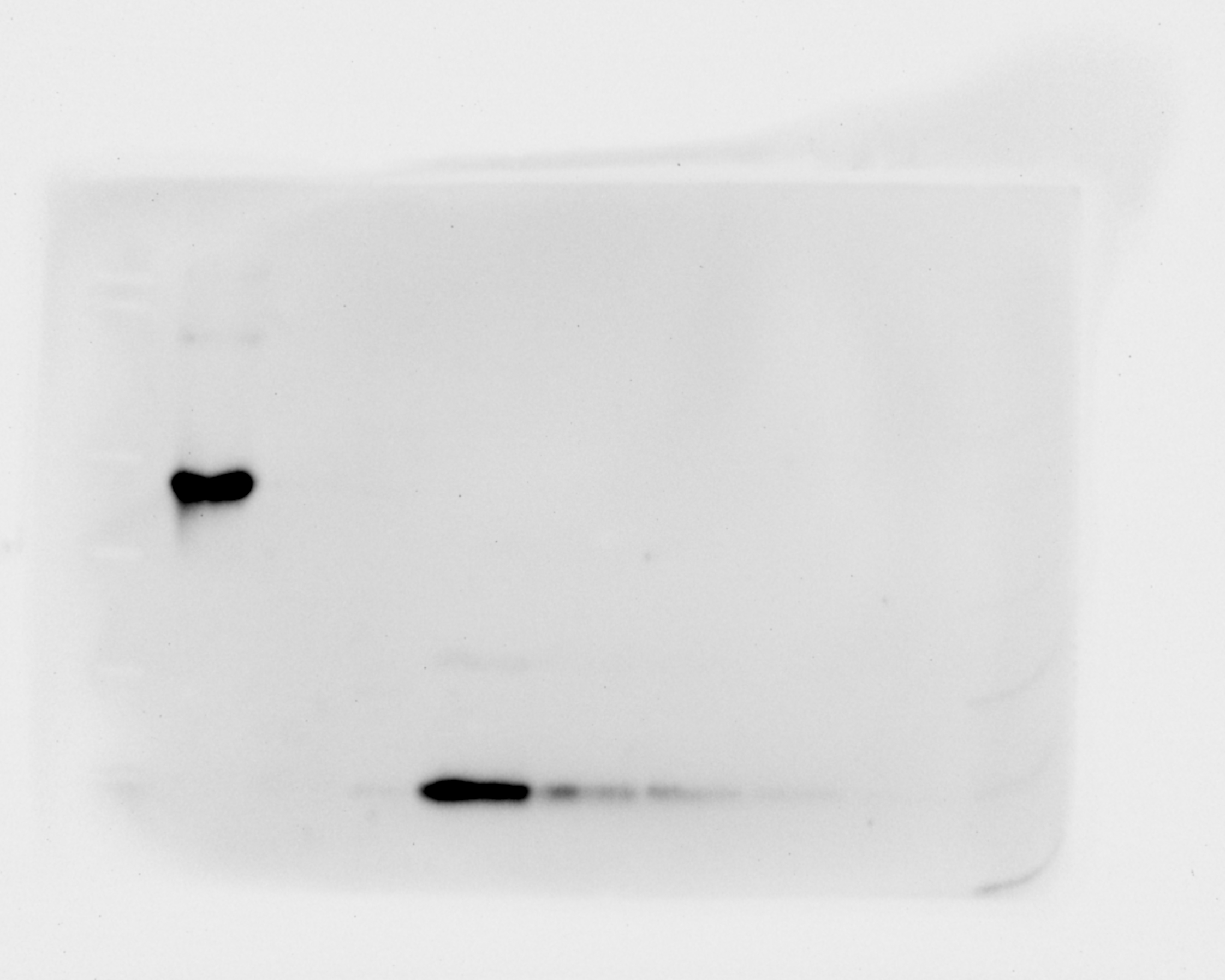

Supplement: S1 Raw files — (ZIP) [file pone.0240617.s006.zip › Archivos Raw/SFigure3B.jpg]

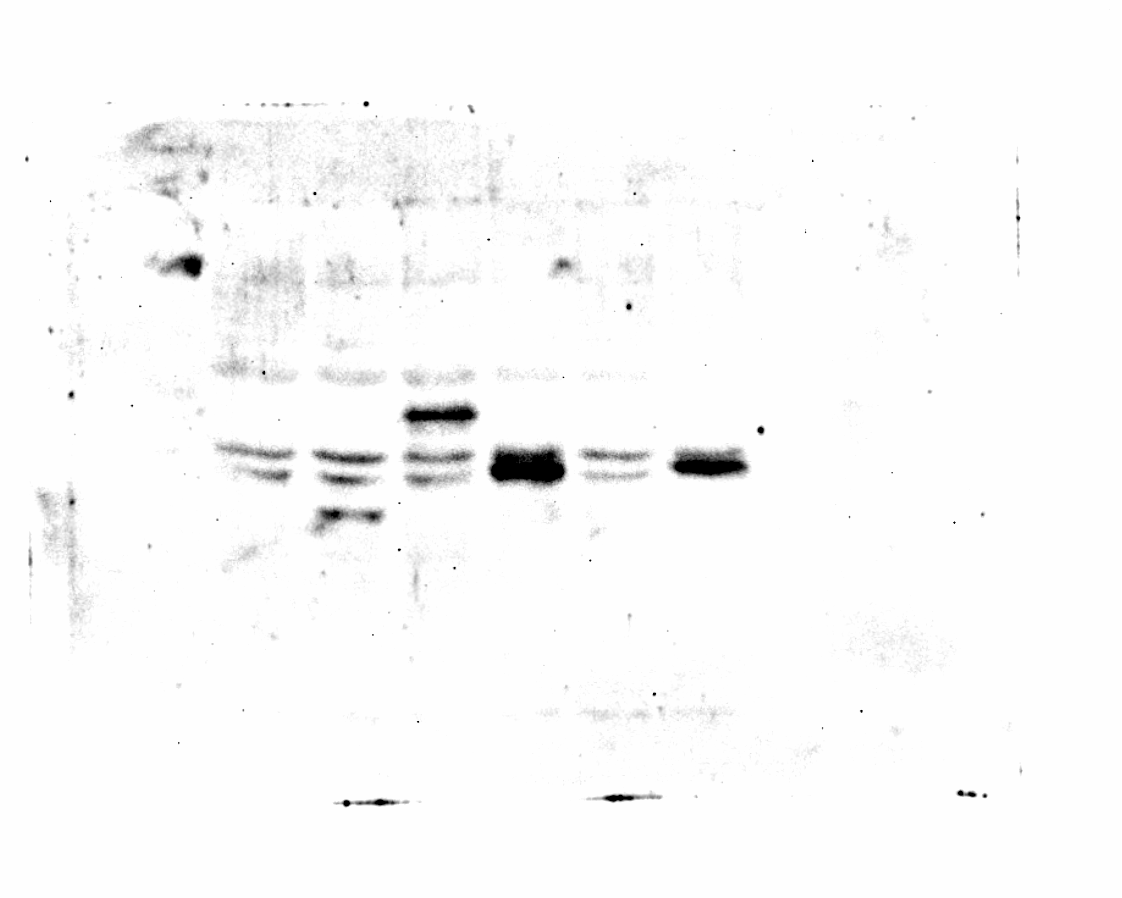

Supplement: S1 Raw files — (ZIP) [file pone.0240617.s006.zip › Archivos Raw/SFigure4.tif]

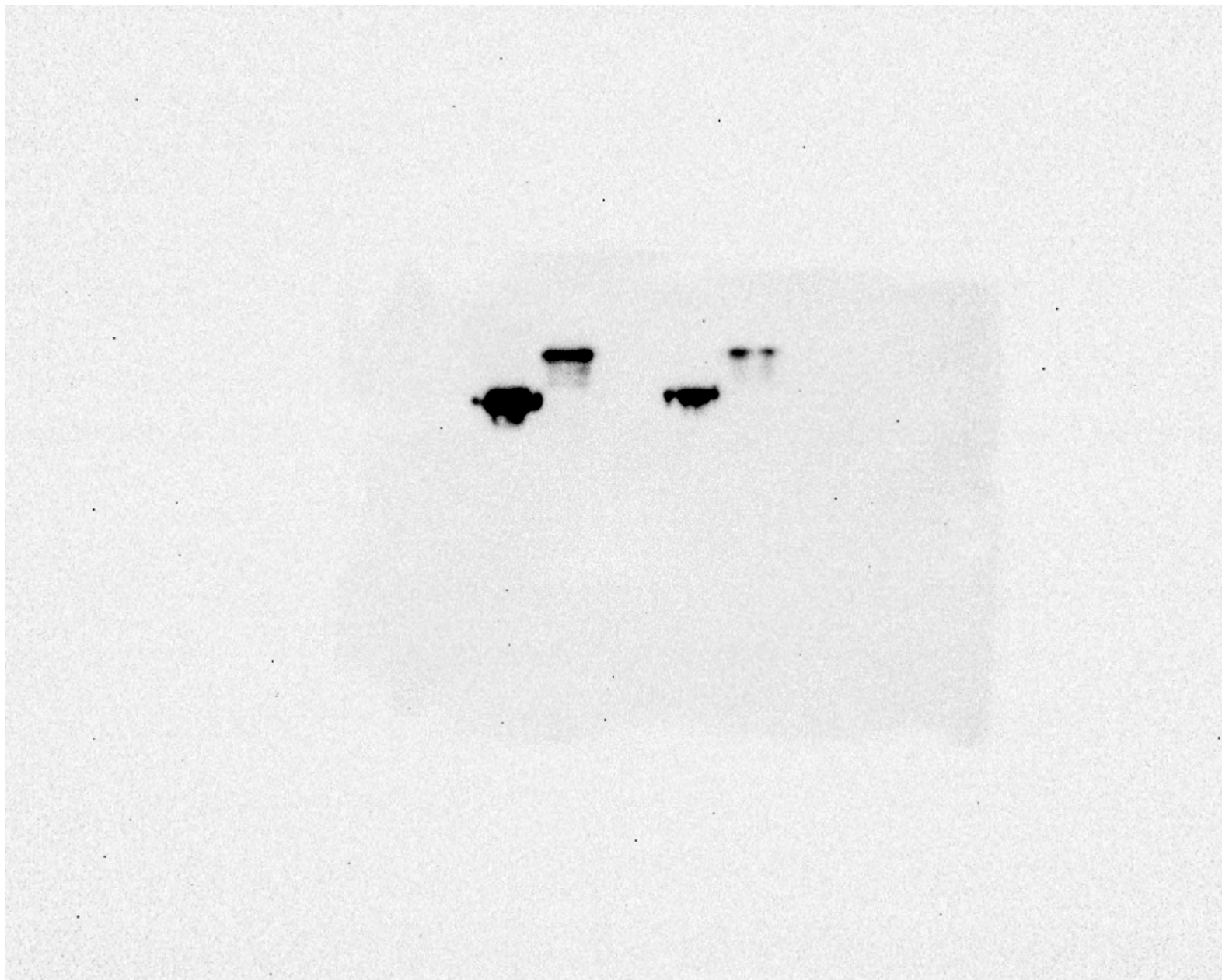

**Fig 4A**

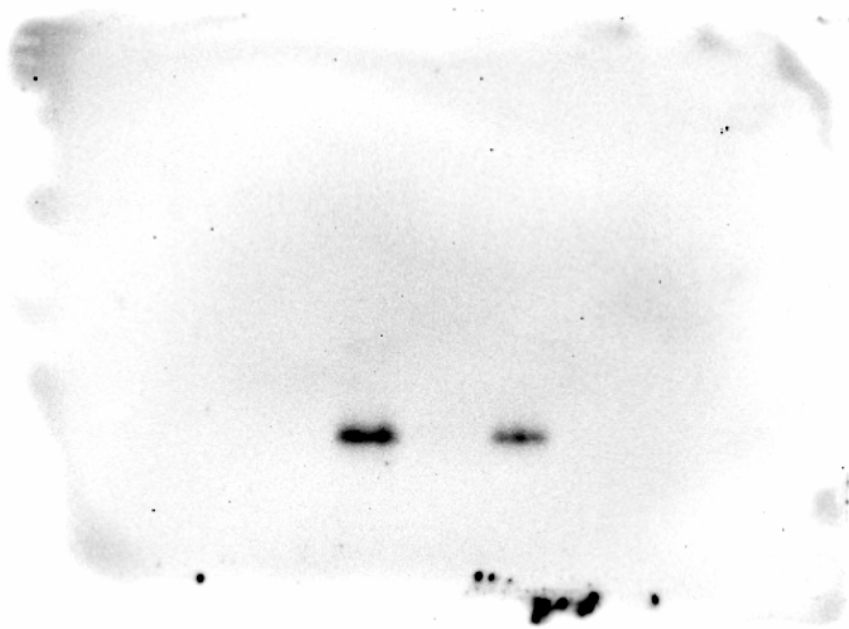

**Fig 4B**

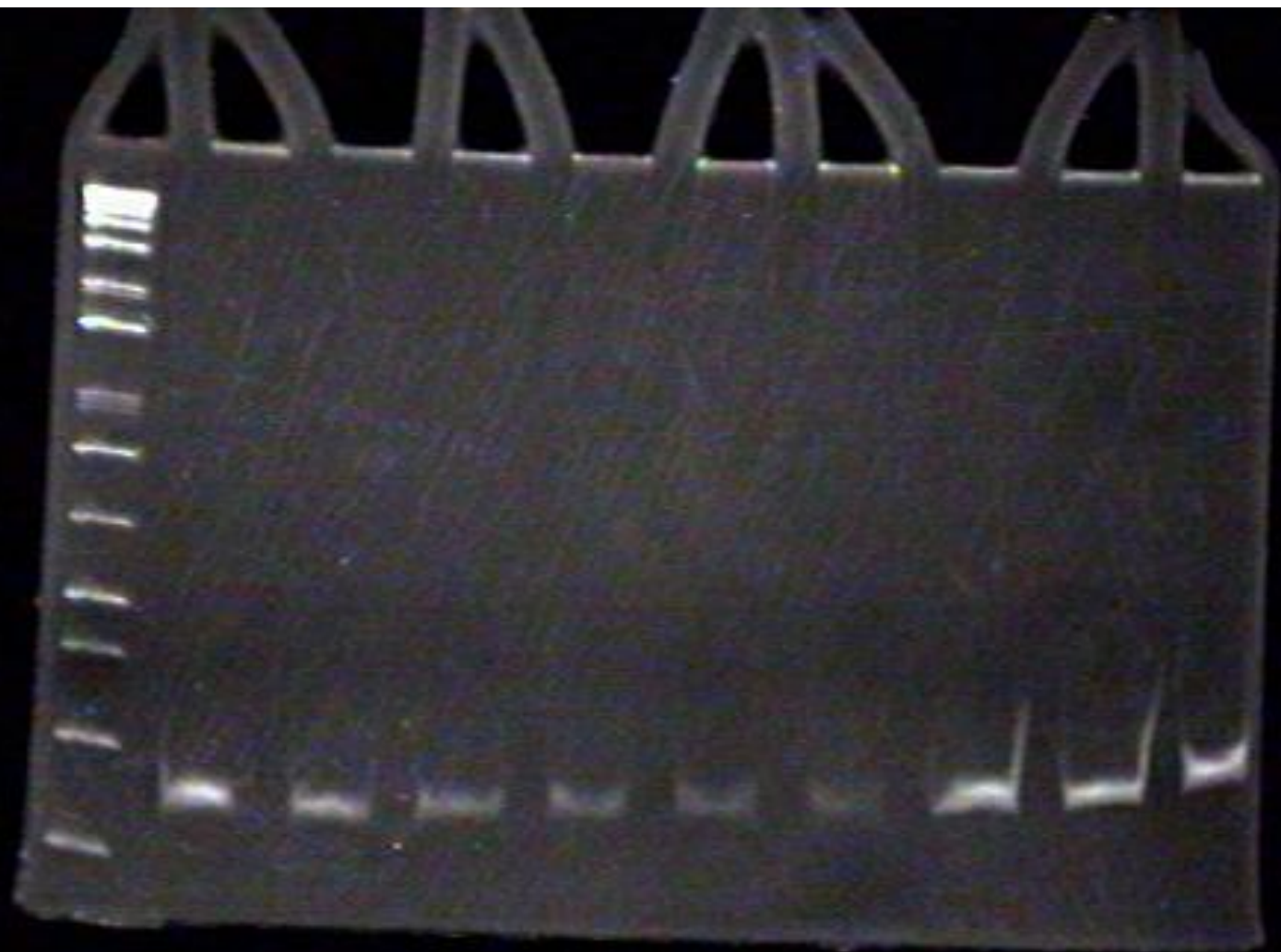

Fig 5A

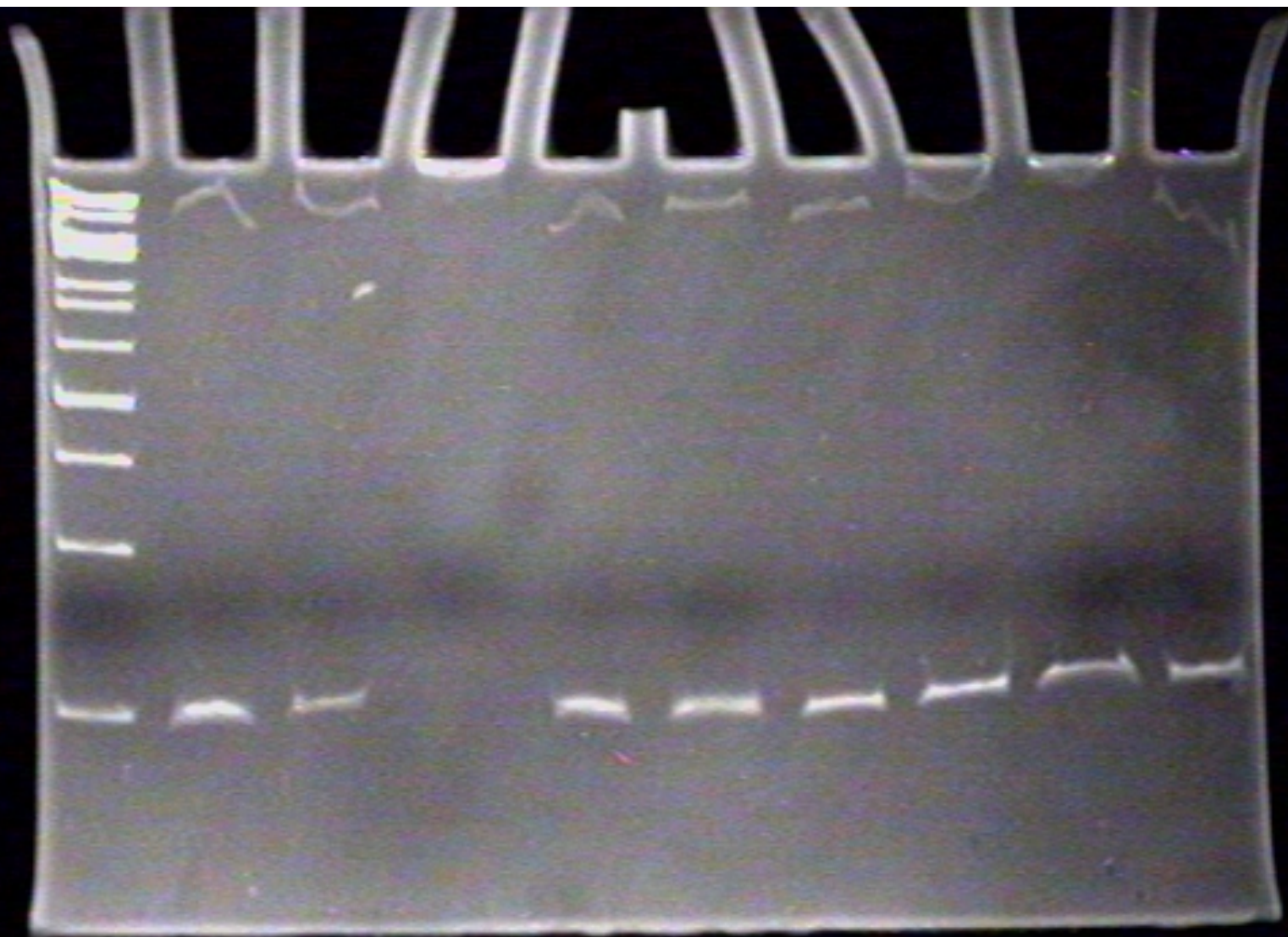

Fig 5B

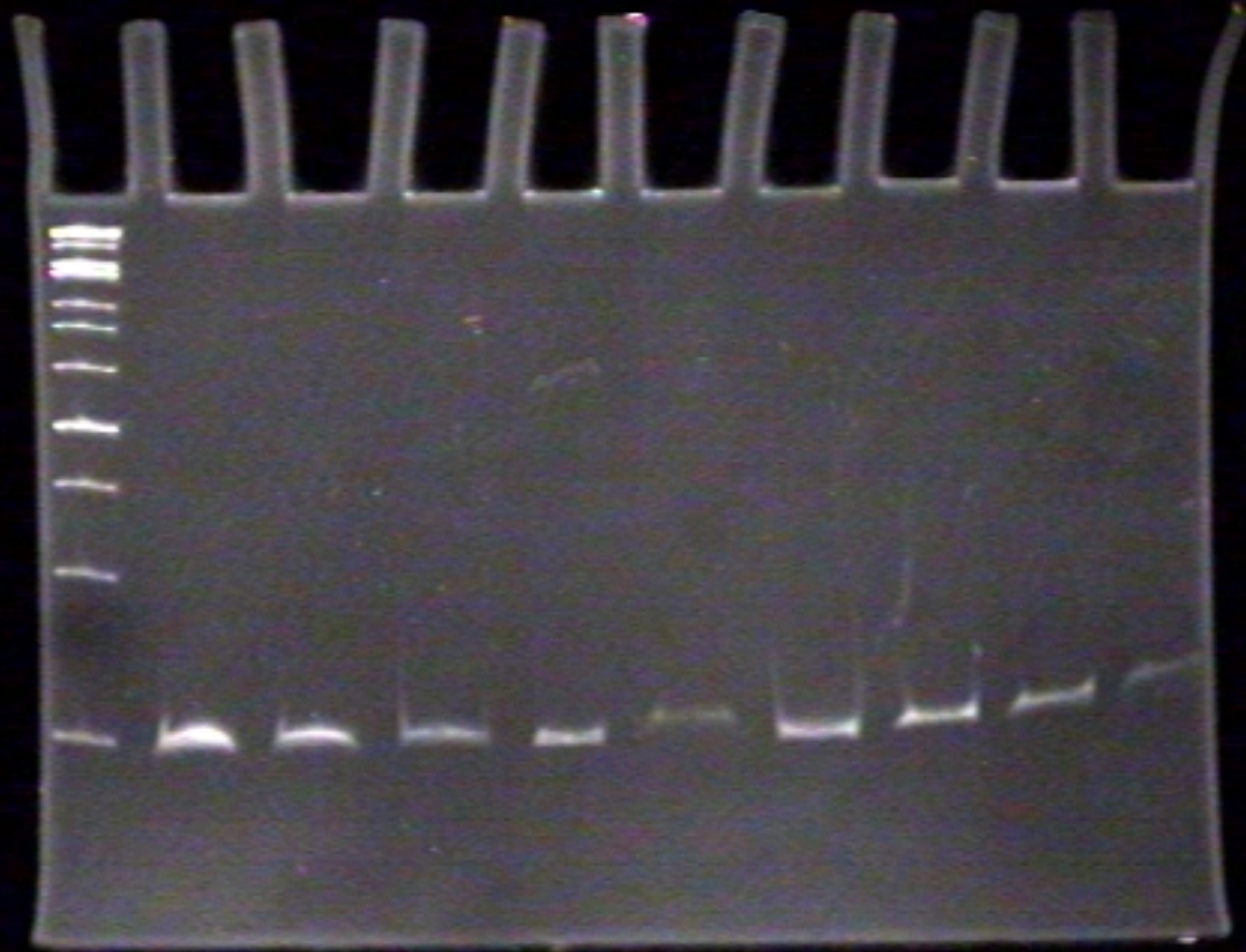

Fig 5C

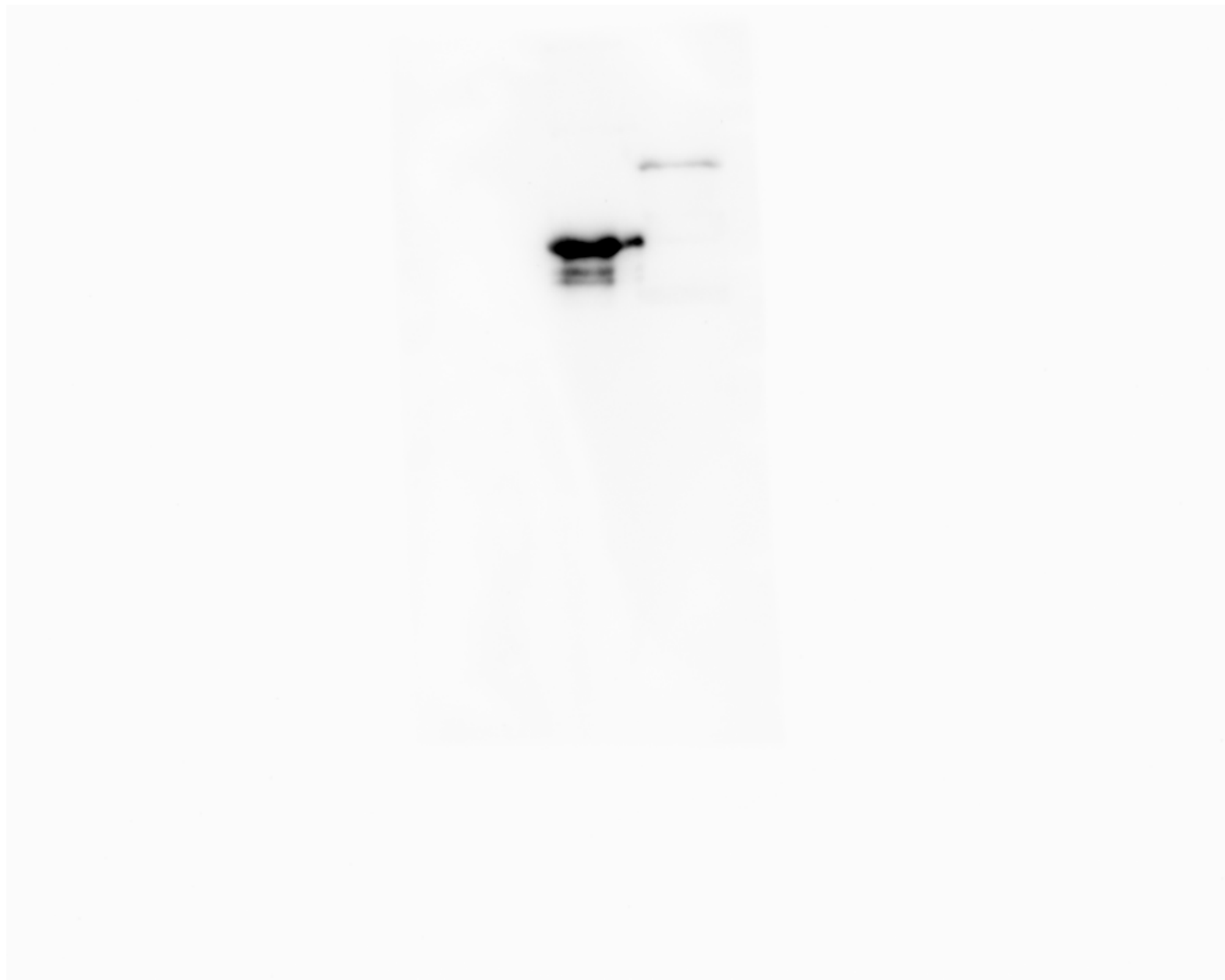

**Fig 7A**

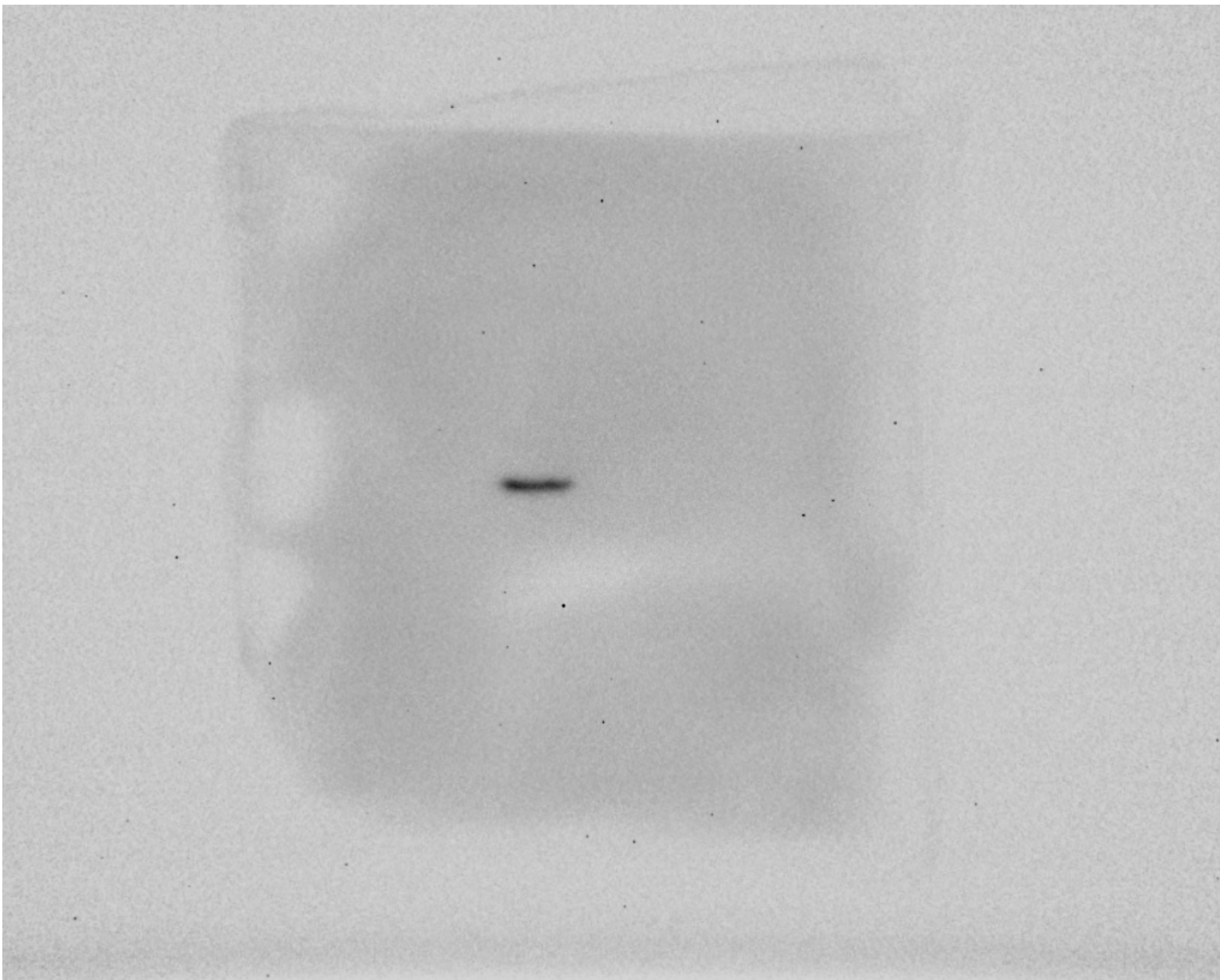

**Fig 7B**

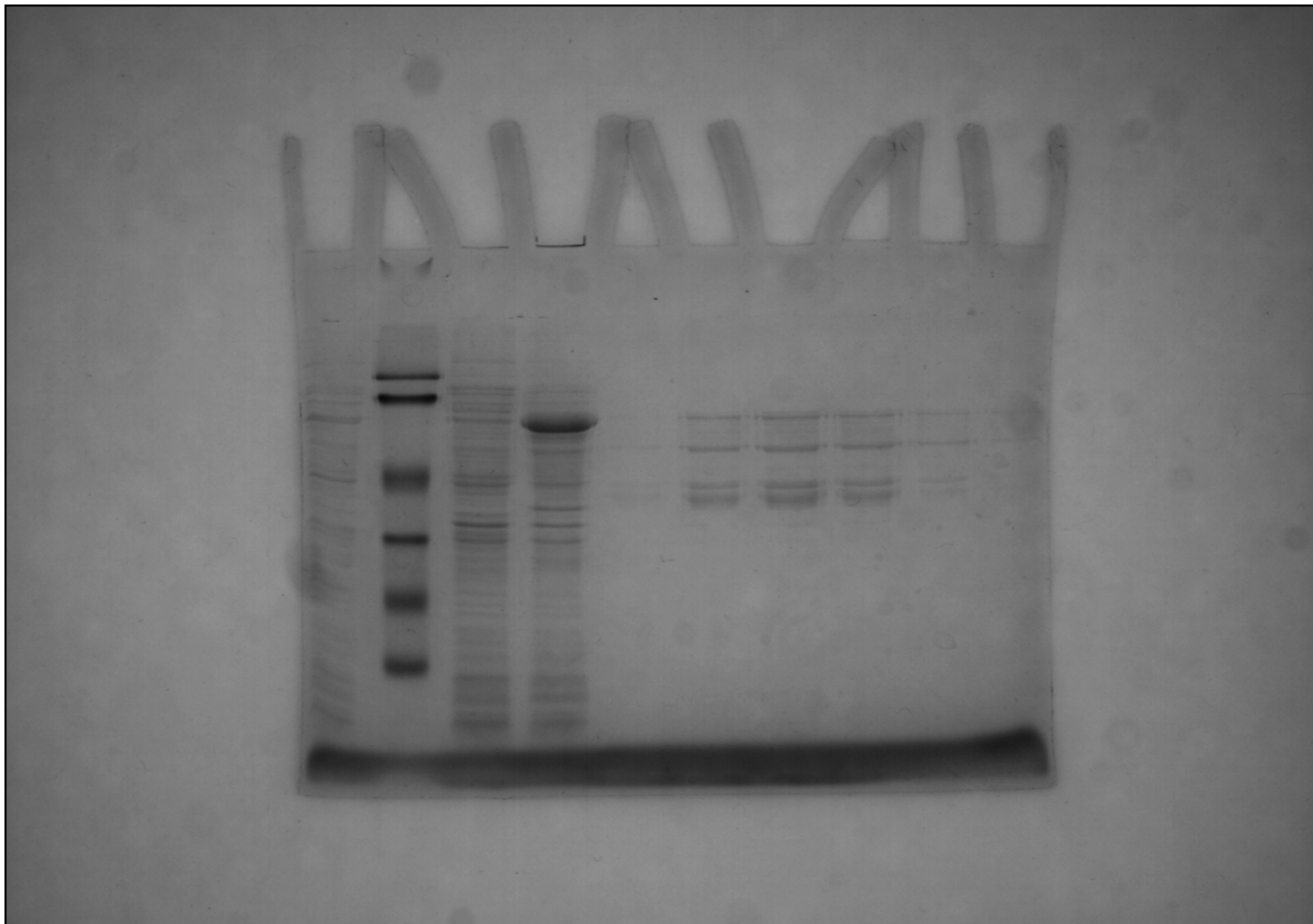

S\_Fig2A

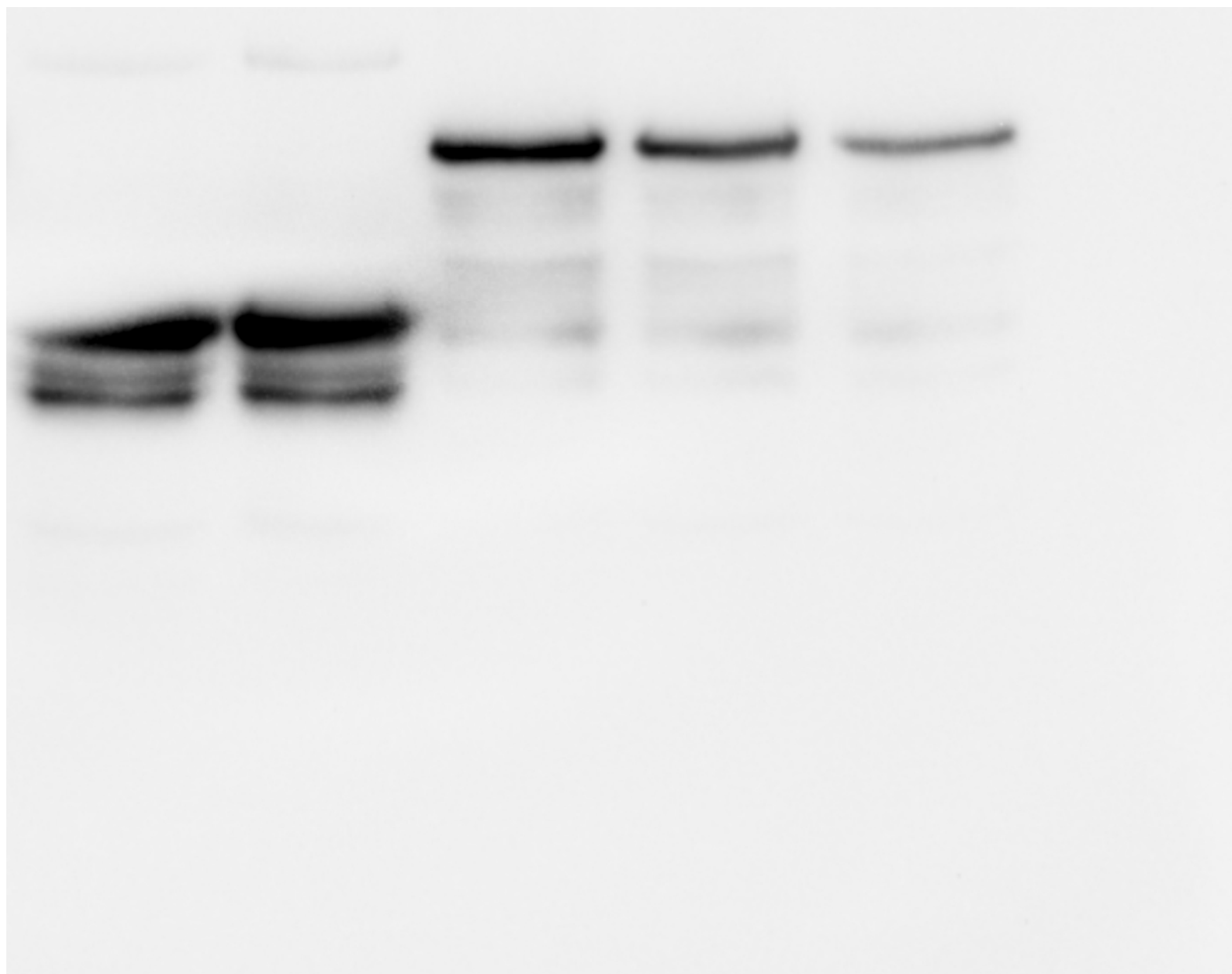

**S\_Fig 2B**

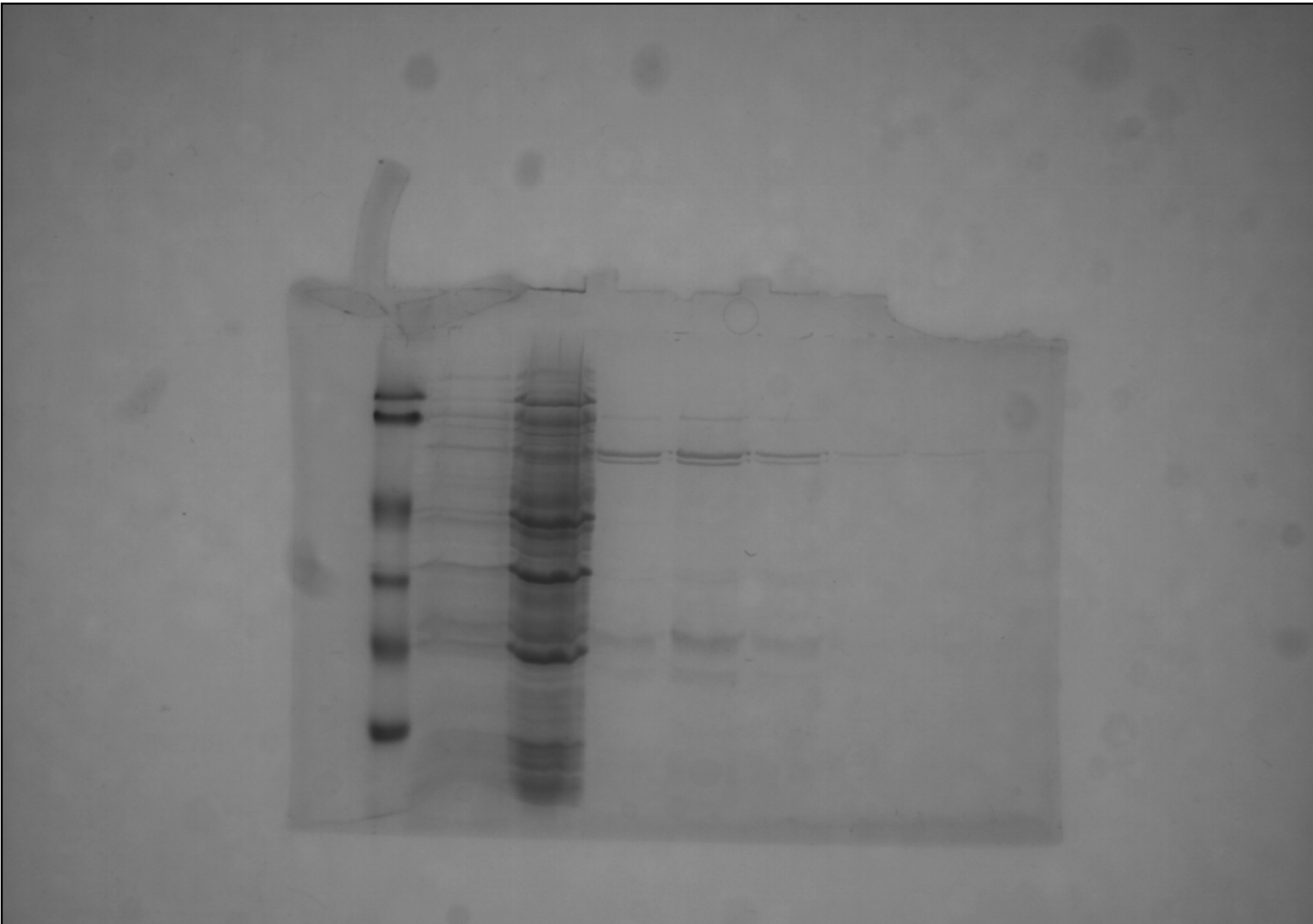

S\_Fig3A

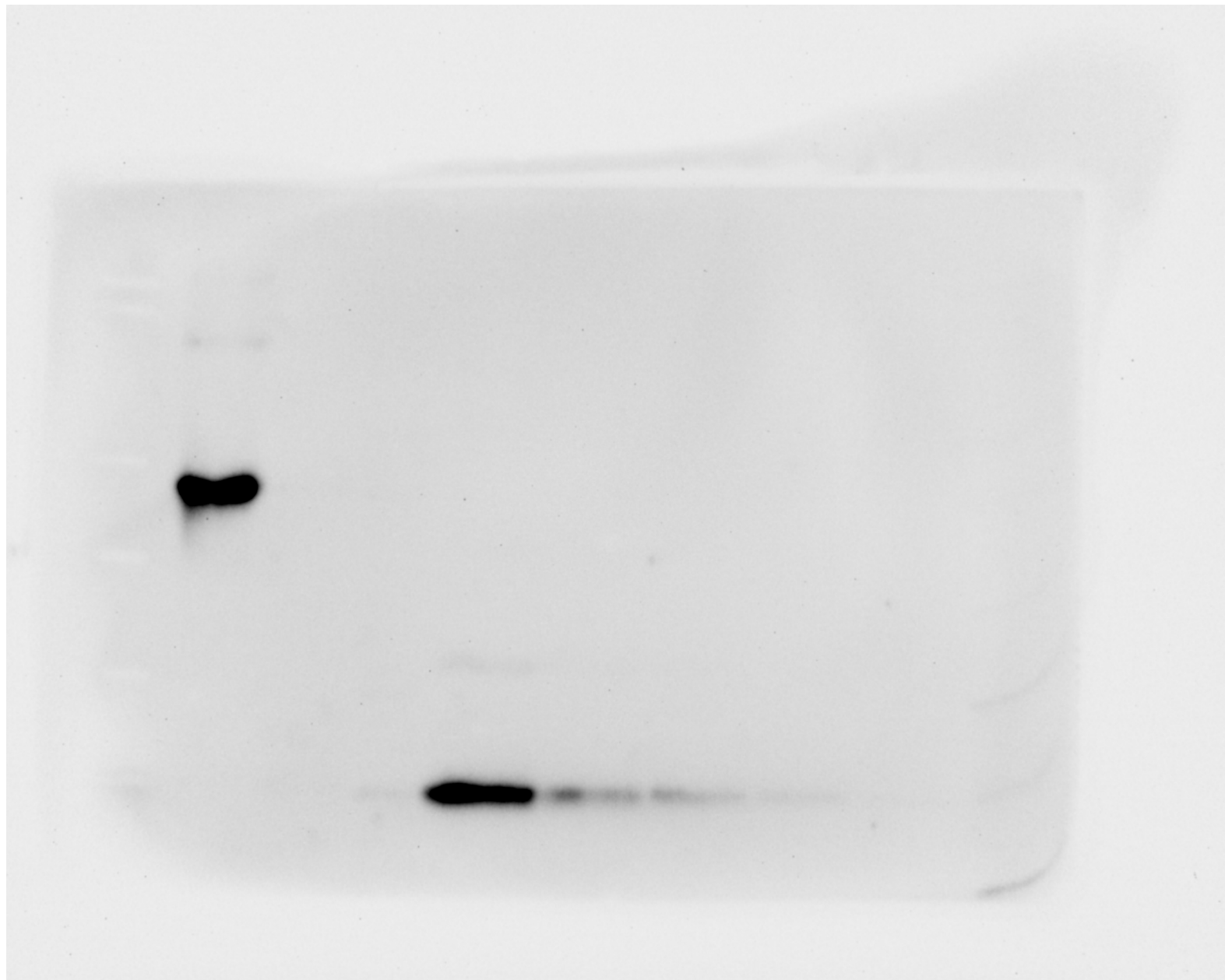

S\_Fig 3B

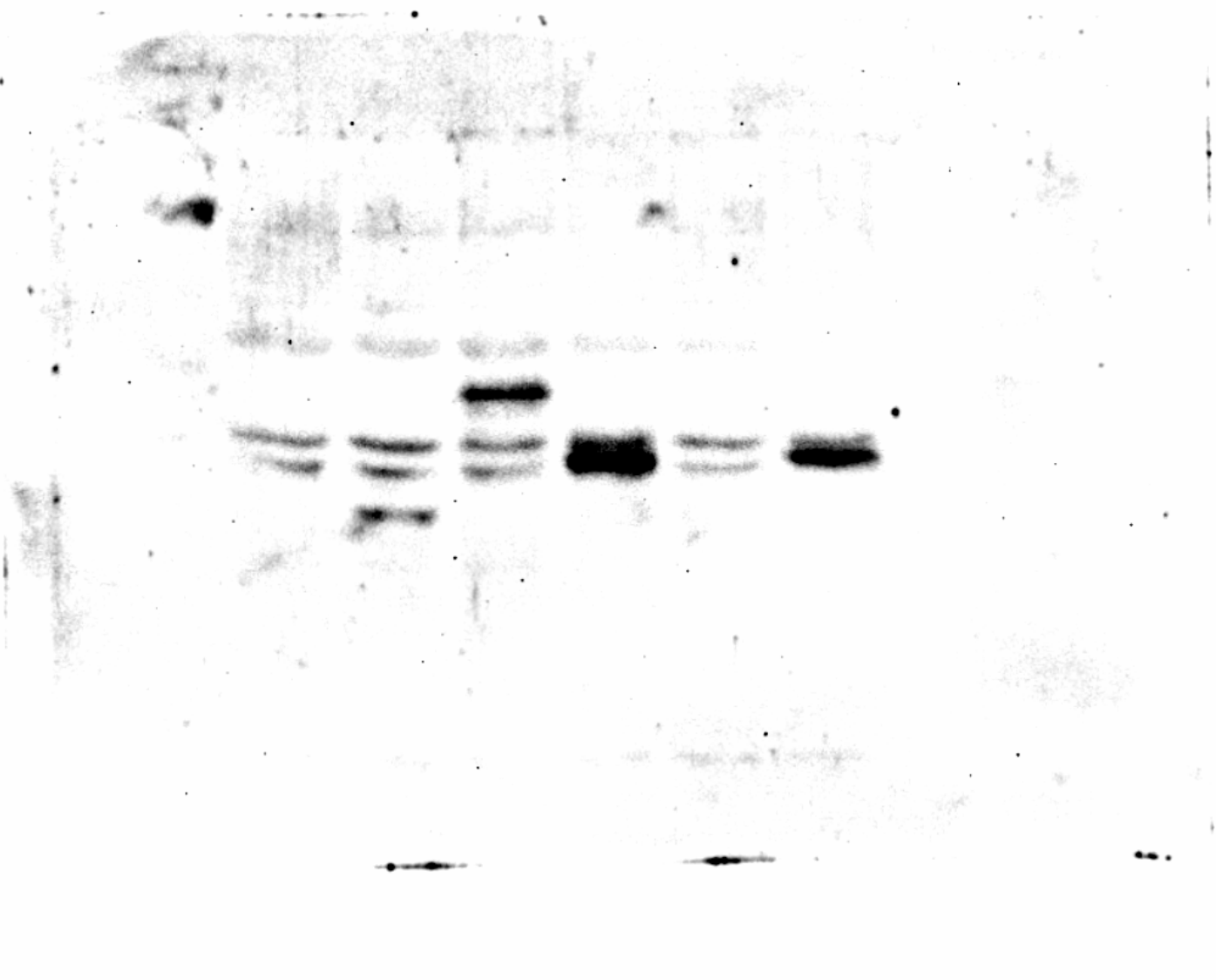

S\_Fig 4

Supplement: S1 Raw pictures — (PDF) [file pone.0240617.s007.pdf]
