# Supplementary material for: The Salmonella Typhimurium InvF-SicA complex is necessary for the transcription of sopB in the absence of the repressor H-NS
Source: PLoS One. 2020 Oct 29;15(10):e0240617. doi: 10.1371/journal.pone.0240617 (PMC7595419; doi:10.1371/journal.pone.0240617)
Supplement: S1 Table — (DOCX) [file pone.0240617.s005.docx]

**S1 Table.** **Plasmids and oligonucleotides used in this study.**

| **Plasmid** | **Description** | **Source*** |
| --- | --- | --- |
| pMPM-T3 | p15A derivative low-copy-number cloning vector, *lac* promoter, Tc^R^ | [1] |
| pT3-InvF | pMPM-T3 derivative expressing InvF from the *lac* promoter | This work |
| pMal-InvF | pMAL-c2X derivative expressing MBP-InvF from a *tac* promoter, Ap^R^ | Virginia Miller, UNC. Personal communication |
| pMal-c2xa | Low copy number plasmid to generate fusions to the *malE* gene for expressing MBP, Ap^R^ | New England Biolabs |
| pTOPO-SicA | pCRTOPO derivative expressing SicA-His6 from the T7 promoter, Km^R^ Ap^R^ | This work |
| pCR2.1TOPO | Cloning vector for PCR products, Km^R^ Ap^R^ | Thermo Scientific |
| psopB-cat1 | pKK232-8 derivative containing a *sopB-cat* transcriptional fusion | [2] |
| philA-cat-410+66 | pKK232-8 derivative containing a *hilA-cat* transcriptional fusion from nucleotides -410 to +66 | [3] |
| pSR658 | ColE1 derivative cloning vector, *lac* promoter, Tc^R^ | [4] |
| pSR659 | p15A derivative low-copy-number cloning vector, *lac* promoter, Ap^R^ | [4] |
| pSR658-InvF | pSR658 derivative expressing LexA_DBDwt_-InvF from the *lac* promoter | This work |
| pSR658-SicA | pSR658 derivative expressing LexA_DBDwt_-SicA from the *lac* promoter | This work |
| pSR659-SicA | pSR659 derivative expressing LexA_DBDmut_-SicA from the *lac* promoter | This work |
| pSR658-HNS | pSR658 derivative expressing LexA_DBDwt_-H-NS from the *lac* promoter | [5] |
| pSR658-HilD | pSR658 derivative expressing LexA_DBDwt_-HilD from the *lac* promoter | [5] |
| pSR659-HilE | pSR659 derivative expressing LexA_DBDmut_-HilE from the *lac* promoter | [5] |
| pET-GlpQ | pET19b derivative with synthetic *glpQ* cloned in phase with 10 histidine codons | [6] |
| **Oligonucleotide** | **Sequence (5’-3’)** | **Source** |
| invF-H3- Rv | GCGAAGCTTAAGAATATGTGTCTTCATTTGTCTGC | This work |
| invF-XH1-Fw | ACCGCTCGAGTTAACTGGTGCTGACAACTATG | This work |
| sicA-Fw | GCGACTCGAGACAGATAACAGGAGTAAGTAATGG | This work |
| sicA-His6-Rv | CGCAAGCTTTTAGTGATGGTGATGGTGATGTTC CTTTTCTTGTTCACTGTGC | This work |
| sopB-RT-Fw | AAG CAG CTT AAT AAC CAG CCC | This work |
| sopB-RT-Rv | ACC GTC CTC ATG CAC ACT CAC | This work |
| Eub338F | ACTCCTACGGGAGGCAGCAG | [7] |
| Eub518R | ATTACCGCGGCTGCTGG | [7] |
| sopB-200-Fw | ATATCTAGACATTTGATGTACCGATCTCCC | This work |
| sopB-Fus-Rv | ATAGGATCCCGTTGTATAAGGTTTTTTGTAGGC | This work |
| sicA-12-Fw | AGCGATGTATTCATTGGGCG | [8] |
| sicA*-*12-Rv | AGCGTGGCGCCTTCACTAAC | [8] |
| sicA-Fw | GCGACTCGAGACAGATAACAGGAGTAAGTAATGG | This work |
| sicA-His6-Rev | CGCAAGCTTTTAGTGATGGTGATGGTGATGTTCCTTTTCTTGTTCACTGTG C | This work |
| fliC-Fw | GCTATCGAGCGTCTGTCTTC | This work |
| fliC-Rv | TCAGCCTGGATGGAGTCGAG | This work |
| lexA-invF-Fw | CCGCTCGAGATGCTAAATACGCAGGAAGTAC | This work |
| lexA-invF-Rv2 | AACTGCAGTATGTGTCTTCATTTGTCTGCC | This work |
| sicA-Lex-Fw | CCGCTCGAGATGGATTATCAAAATAATGTCAGCGAAG | This work |
| sicA-Lex-Rv2 | AACTGCAGTTATTCCTTTTCTTGTTCACTGTGC | This work |
| sicA-Lex-Rv3 | GGGGTACCTTATTCCTTTTCTTGTTCACTGTG | This work |

*. References included in this table are listed below.

**References**

1. Mayer MP. 1995. A new set of useful cloning and expression vectors derived from pBlueScript. Gene 163(1): 41-46. doi: 10.1016/0378-1119(95)00389-n.
2. Bustamante VH, Martínez LC, Santana FJ, Knodler LA, Steele-Mortimer O, Puente JL. 2008. HilD-mediated transcriptional cross-talk between SPI-1 and SPI-2. Proc Natl Acad Sci USA. 105(38): 14591-14596. doi: 10.1073/pnas.0801205105.
3. Pérez-Morales D, Banda MM, Chau NYE, Salgado H, Martínez-Flores I, Ibarra JA, et al. 2017. The transcriptional regulator SsrB is involved in a molecular switch controlling virulence lifestyles of *Salmonella*. PLoS Pathog. 13(7): e1006497. doi: 10.1371/journal.ppat.1006497.
4. Daines DA, RP Silver. 2000. Evidence for multimerization of neu proteins involved in polysialic acid synthesis in *Escherichia coli* K1 using improved LexA-based vectors. J Bacteriol 182: 5267-5270. doi: 10.1128/jb.182.18.5267-5270.2000.
5. Paredes-Amaya CC, G Valdés-García, VR Juárez-González, E Rudiño-Piñera, BH Bustamante. 2018. The Hcp-like protein HilE inhibits homodimerization and DNA binding of the virulence-associated transcriptional regulator HilD in *Salmonella*. J Biol Chem 293: 6578-6592. doi: 10.1074/jbc.RA117.001421.
6. Vázquez-Guerrero E, Adan-Bante NP, Mercado-Uribe MC, Hernández-Rodríguez C, Villa-Tanaca L, Lopez JE, et al. 2019. Case report: A retrospective serological analysis indicating human exposure to tick-borne relapsing fever spirochetes in Sonora, Mexico. PLoS Negl Trop Dis. 13(4): e0007215. doi: 10.1371/journal.pntd.0007215.
7. Guo X, Xia X, Tang R, Wang K. 2008. Real-time PCR quantification of the predominant bacterial divisions in the distal gut of Meishan and Landrace pigs. Anaerobe. 14(4): 224-228. doi: 10.1016/j.anaerobe.2008.04.001.
8. Darwin KH, Miller VL. 2001. Type III secretion chaperone-dependent regulation: activation of virulence genes by SicA and InvF in *Salmonella* Typhimurium. EMBO J. 20(8): 1850-1862. doi: 10.1093/emboj/20.8.1850.
